# HRMS-Targeted-DIA methodology for quantification of wastewater-borne pollutants in surface water

**DOI:** 10.1016/j.mex.2023.102093

**Published:** 2023-02-24

**Authors:** Olga Gómez-Navarro, Francesc Labad, Diana P Manjarrés-López, Sandra Pérez, Nicola Montemurro

**Affiliations:** ONHEALTH, Department of Environmental Chemistry, Institute of Environmental Assessment and Water Research (IDAEA-CSIC), Jordi Girona 18-26, Barcelona 08034, Spain

**Keywords:** Contaminants of emerging concern, Pharmaceuticals, High-resolution mass spectrometry, Multi-layered SPE, Streams, CECs targeted method for surface water

## Abstract

A robust method was developed for the quantification of popular and highly occurrence contaminants of emerging concern from wastewater treatment plant effluents and is explained in detail. A homemade multi-layered and multi-sorbent solid phase extraction (SPE) cartridge was used to cover the wide range of polarities of the selected contaminants. A non-discriminant elution protocol was also applied. Liquid chromatography coupled to a high-resolution mass spectrometer (HRMS) Q-Exactive Orbitrap system was used for the separation and detection of the contaminants. A targeted data independent acquisition (DIA) mode with an inclusion list with the exact mass, retention time window and collision energy was tried for the first time obtaining good sensitivity, selectivity and high quality MS2 product ions.

• 116 compounds of a wide-scope of polarities and physic-chemical properties were validated using a surface water pool matrix.
• SPE followed by LC—HRMS with a targeted DIA was used for the method validation at three concentration levels 5, 50, 500 μg l^-1^ in extract.
• Good recoveries were obtained between 70 and 120% for the majority of the selected contaminants. Matrix effect, precision, and linearity were also evaluated and results proved the suitability for the method application.

## Graphical abstract

**Figure:**
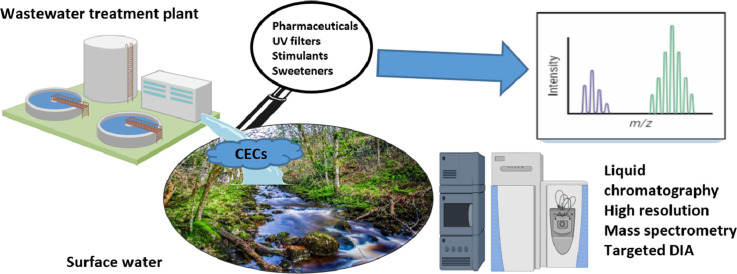

Specifications table

**Table:** 

Subject area:	Environmental Science
More specific subject area:	*Environmental Analytical Chemistry – Organic Contaminants*
Name of your method:	*CECs targeted method for surface water*
Name and reference of original method:	*This analytical method is based on the method described by Enelton Fagnani, Nicola Montemurro and Sandra Pérez (2022). Multilayered solid phase extraction and ultra performance liquid chromatographic method for suspect screening of halogenated pharmaceuticals and photo-transformation products in freshwater - comparison between data-dependent and data-independent acquisition mass spectrometry*, Journal of Chromatography A https://doi.org/10.1016/j.chroma.2021.462760
Resource availability:	*NA*

## Method details

### Background

Water is a basic need that is necessary in our day to day life and is impossible to do without, thus its constant use. After its use, different treatments are applied to ensure its return to the ecosystem of origin (back to rivers, sea or ocean) or for its future reuse. Wastewater is usually managed through wastewater treatment plants, (WWTPs), especially in developed countries, and it may contain several contaminants such as nutrients, metals, parasites and contaminants of emerging concern (CECs) [1]. However, it has been demonstrated that WWTPs are not sufficiently efficient in their removal and many wastewater-borne pollutants are continuously being released back into the environment. Through WWTPs, wastewater-borne pollutants are introduced into surface waters, where sometimes the wastewater effluent can even dominate those streams and rivers where low flow conditions are present. Wastewater-borne pollutants enclose various types of contaminants and are of growing concern. New pollutants are continuously detected and many compounds with different physicochemical properties and different polarities are found to be detected after the WWTP discharge [2,3]. However, discharge guidelines and standards are not always available, especially concerning most pharmaceuticals or personal care products even though their use is continuously increasing. Therefore, a targeted method was developed to be able to track and control the continuous release of these compounds by WWTPs as they should be monitored due to their potential undesirable effect on the environment.

In this work, an extraction method based on a multi-layered and multi-sorbent solid phase extraction was combined with a non-discriminant elution protocol to cover a wide range of different contaminants of emerging concern that end up in rivers mainly derived from WWTPs effluent. To study the method's effectiveness, the method was validated for a total of 116 compounds in surface water samples.

### Chemicals and reagents

The analytes included for this targeted analysis were selected concerning their environmental occurrence as well as their presence in the list of priority substances in surface water from the EU Water Framework Directive [4] and previous group experience [5], [6], [7]. The list of selected compounds for a total of 116 compounds with a wide range of polarities (Log P ranging from -2.16 to 5.62) and different physicochemical properties are given in Table 1 including the name of the target compound, CAS number, molecular formula, monoisotopic mass, Log P and classification of compounds. Several classes of CECs are included in this list, mainly pharmaceuticals, UV filters, stimulants, sweeteners and some critical metabolites and transformation products (TPs). Extended details of the compounds are also included in Table SM 1. All analytical reference standards were of high purity and purchased from Sigma Aldrich (St. Louis, MO U.S). Isotopically labelled compounds used as surrogates for calibration purposes were purchased from Cerilliant (Sigma Aldrich, St. Lous, MO, U.S), Santa Cruz Biotechnology (Dallas, TX, US.), or Toronto Research Chemicals (Toronto, ON, Canada) and are listed in Table 5 in the supplementary material. For standard, samples, and calibration curve preparation, pure water, methanol (MeOH), acetonitrile (ACN) and ethyl acetate (EtOAc) of high-performance liquid chromatography (HPLC) grade were obtained from J.T. Backer (Deventer, The Netherlands). Formic acid (≥98.0%, ACS grade), ammonium acetate (AcNH_4_), and concentrated ammonia solution (NH_4_OH) (29.0%, ACS grade) were supplied by Sigma-Aldrich. Ammonium fluoride (NH_4_F) was purchased from Fisher Chemical (Fisher Scientific SL, Madrid, Spain). The solid phase extraction (SPE) sorbents for the homemade SPE cartridges preparation were Oasis HLB, WAX, and WCX (60 µm particle size) purchased from Waters Corp., (Milford, MA, USA), while Bond Elut PPL (63–150 µm particle size) was purchased from Agilent Technologies Inc. (Santa Clara, CA, USA). Empty 6 mL polypropylene cartridges were purchased from Phenomenex Inc. (Torrance, CA, USA).

**Table 1:** Target compounds name, CAS number, molecular formula, monoisotopic mass, Log P and compound classification.

**Compound name**	**CAS number**	**Molecular Formula**	**Monoisotopic mass**	Log P*	**Classification**
1,7 Dimethylxanthine	611–59–6	C7H8N4O2	180.0647	0.24	Stimulant
17-α Ethynilestradiol	57–63–6	C20H24O2	296.1776	3.9	Hormone
1H-Benzotriazole	95–14–7	C6H5N3	119.0483	1.3	Anticorrosive agent
2.4-Dihydroxybenzophenone (BP1)	131–56–6	C13H10O3	214.0630	3.48	UV filter
2.2′.4.4′-Tetrahydroxybenzophenone (BP2)	131–55–5	C13H10O5	246.0528	2.8	UV filter
4-Hydroxidiclofenac	64,118–84–9	C14H11Cl2NO3	311.0116	3.96	Anti-inflammatory TP / Metabolite
4-Nitro Sulfamethoxazole	29,699–89–6	C10H9N3O5S	283.0263	1.56	Antibiotic TP
5-Desamino-5 oxo 2,5 Lamotrigine	252,186–78–0	C9H6Cl2N4O	255.9919	2.24	Antiepileptic TP
5-methyl-1 H Benzotriazole	136–85–6	C7H7N3	133.0640	1.81	Anticorrosive agent
5-nitro-Diclofenac	66,505–80–4	C14H10Cl2N2O4	340.0018	4.26	Anti-inflammatory TP
6a-Methylprednisolone	83–43–2	C22H30O5	374.2093	1.56	Anti-inflammatory
Acesulfame	33,665–90–6	C4H5NO4S	162.9939	-0.55	Sweetener
Acetaminophen	103–90–2	C8H9NO2	151.0633	0.91	Analgesic
Acridone	578–95–0	C13H9NO	195.0684	4.2	Anticonvulsant TP
Adamantan-1-amine (Amantadine)	768–94–5	C10H17N	151.1361	1.47	Antiviral
Alprazolam	28,981–97–7	C17H13ClN4	308.0829	3.02	Anxiolytic
Atenolol	29,122–68–7	C14H22N2O3	266.1631	0.43	β-blocker agent
Azithromycin	83,905–01–5	C38H72N2O12	748.5085	2.44	Antibiotic
Benzoylecgonine	519–09–5	C16H19NO4	289.1314	-0.59	Metabolite
B-estradiol	50–28–2	C18H24O2	272.1776	4.01	Hormone
Bezafibrate	41,859–67–0	C19H20ClNO4	361.1081	3.99	Lipid regulator
Bisphenol-A	80–05–7	C15H16O2	228.1150	4.04	Plasticizer
Bromazepam	1812–30–2	C14H10BrN3O	315.0007	2.54	Anxiolytic
Caffeine	58–08–2	C8H10N4O2	194.0804	-0.55	Stimulant
Carazolol	57,775–29–8	C18H22N2O2	298.1681	2.71	β-blocker agent
Carbamazepine	298–46–4	C15H12N2O	236.095	2.77	Anticonvulsant
Carbamazepine-10,11-epoxide	36,507–30–9	C15H12N2O2	252.0899	1.97	Anticonvulsant TP/ Metabolite
Carisoprodol	78–44–4	C12H24N2O4	260.1736	1.92	Muscle relaxant
Chloramphenicol	56–75–7	C11H12Cl2N2O5	322.0123	0.88	Antibiotic
Citalopram	59,729–33–8	C20H21FN2O	324.1638	3.76	Antidepressant
Clarithromycin	81,103–11–9	C38H69NO13	747.4769	3.24	Antibiotic
Climbazole	38,083–17–9	C15H17ClN2O2	292.0979	4.34	Antibiotic
Clofibric Acid	882–09–7	C10H11ClO3	214.0397	2.9	Lipid regulators
Cocaethylene	529–38–4	C18H23NO4	317.1627	2.64	Metabolite
Cocaine	50–36–2	C17H21NO4	303.1471	2.28	Stimulant
Codeine	76–57–3	C18H21NO3	299.1521	1.34	Analgesic
Cotinine	486–56–6	C10H12N2O	176.095	0.21	Stimulant
Crotamiton	483–63–6	C13H17NO	203.131	3.09	anti-itching drug
Cyclamate	100–88–9	C6H13NO3S	179.0616	-1.61	Sweetener
Diazepam	439–14–5	C16H13ClN2O	284.0717	3.08	Anxiolytic
Diclofenac	15,307–86–5	C14H11Cl2NO2	295.0167	4.26	Anti-inflammatory
Diltiazem	42,399–41–7	C22H26N204S	414.1613	2.73	Antihypertensive
Estriol	50–27–1	C18H24O3	288.1725	2.67	Hormone
Estrone	53–16–7	C18H22O2	270.162	4.31	Hormone
Ezetimibe	163,222–33–1	C24H21F2NO3	409.149	4.56	Lipid regulator
Fipronil	120,068–37–3	C12H4Cl2F6N4OS	435.9387	4.49	Insecticide
Fipronil desulfinyl	205,650–65–3	C12H4Cl2F6N4	387.9717	4.53	Insecticide TP
Fipronil sulfide	120,067–83–6	C12H4Cl2F6N4S	419.9438	5.62	Insecticide TP
Fipronil sulfone	120,068–36–2	C12H4Cl2F6N4O2S	451.9336	4.6	Insecticide TP
Florfenicol	73,231–34–2	C12H14Cl2FNO4S	357.0005	0.67	Antibiotic
Fluconazole	86,386–73–4	C13H12F2N6O	306.1041	0.56	Antifugal
Flumequine	42,835–25–6	C14H12FNO3	261.0801	2.42	Antibiotic
Fluticasone propionate	80,474–14–2	C25H31F3O5S	500.1844	4.86	Anti-inflammatory
Furazolidone	67–45–8	C8H7N3O5	225.0386	0.87	Antibiotic
Furosemide	54–31–9	C12H11ClN2O5S	330.0077	1.75	Diuretic
Gemfibrozil	25,812–30–0	C15H22O3	250.1569	4.39	Lipid regulator
Hydrochlorothiazide	58–93–5	C7H8ClN3O4S2	296.9645	-0.58	Diuretic
Hydroxychloroquine	118–42–3	C18H26ClN3O	335.1764	1.26	Antimalarial
Hyoscine (scopolamine)	51–34–3	C17H21NO4	360.2175	-1.94	Anticholinergic
Ibuprofen	15,687–27–1	C13H18O2	206.1307	3.84	Anti-inflammatory
Indomethacin	53–86–1	C19H16ClNO4	357.0768	3.53	Anti-inflammatory
Irbesartan	138,402–11–6	C25H28N6O	428.2325	4.47	Antihypertensive
Irgasan (Triclosan)	3380–34–5	C12H7Cl3O2	287.9512	4.98	Antimicrobial
Ketoprofen	22,071–15–4	C16H14O3	254.0943	3.61	Anti-inflammatory
Lamotrigine	84,057–84–1	C9H7Cl2N5	255.0079	1.93	Antiepileptic
Lamotrigine-N2-oxide	136,565–76–9	C9H7Cl2N5O	271.0028	1.76	Antiepileptic TP
Lidocaine	137–58–6	C14H22N2O	234.1732	2.84	Anesthetic
Lincomycin	154–21–2	C18H34N2O6S	406.2138	-0.32	Antibiotic
Lorazepam	846–49–1	C15H10Cl2N2O2	320.0119	4.06	Anxiolytic
Losartan	114,798–26–4	C22H23ClN6O	422.1622	4.06	Antihypertensive
Mefenamic Acid	61–68–7	C15H15NO2	241.1103	5.4	Anti-inflammatory
Meprobamate	57–53–4	C9H18N2O4	218.1267	0.93	Anxiolytic
Methadone	76–99–3	C21H27NO	309.2093	5.01	treatment of addiction
Metoprolol	37,350–58–6	C6H9N3O3	171.0644	1.76	β-blocker agent
Metronidazole	443–48–1	C15H25NO3	267.1834	-0.46	Antibacterial
Midazolam	59,467–70–8	C18H13ClFN3	325.0782	3.97	Anxyiolitic
Morphine	57–27–2	C17H19NO3	285.1365	0.9	opioid
N-acetyl sulfamethoxazole	21,312–10–7	C12H13N3O4S	295.0627	0.86	Antibiotic TP / Metabolite
Nalidixic acid	389–08–2	C12H12N2O3	232.0848	1.01	Antibiotic
Naproxen	22,204–53–1	C14H14O3	230.0943	2.99	Anti-inflammatory
Neotame	165,450–17–9	C20H30N2O5	378.2155	-0.08	Sweetener
N-Methyl lamotrigine	1,373,243–86–7	C10H9Cl2N5	269.0235	3.74	Antiepileptic TP/ Metabolite
O-desmethylvenlafaxine	93413dic-62–8	C16H25NO2	263.1885	2.29	Metabolite
Oseltamivir	196,618–13–0	C16H28N2O4	312.2049	1.16	Antiviral
Oxazepam	604–75–1	C15H11ClN2O2	286.0509	2.92	Anxiolytic
Paroxetine	61,869–08–7	C19H20FNO3	329.1427	3.15	Antidepressant
Pentobarbital	76–74–4	C11H18N2O3	226.1317	1.89	Anxiolytic
Phenytoin	57–41–0	C15H12N2O2	252.0899	2.15	Anticonvulsant
Primidone	125–33–7	C12H14N2O2	218.1055	1.12	Anticonvulsant
Propranolol	525–66–6	C16H21NO2	259.1572	2.58	β-blocker agent
Propyphenazone	479–92–5	C14H18N2O	230.1419	2.35	Analgesic
Rosuvastatin	287,714–41–4	C22H28FN3O6S	481.1683	1.92	Anti-inflammatory
Saccharin	81–07–2	C7H5NO3S	182.9990	0.45	Sweetener
Salicylic acid	69–72–7	C7H6O3	138.0317	1.98	Anti-inflammatory
Sertraline	79,617–96–2	C17H17Cl2N	305.0738	5.15	Antidepressant
Sitagliptin	486,460–32–6	C16H15F6N5O	407.1181	1.26	Antidiabetic
Sotalol	3930–20–9	C12H20N2O3S	272.1195	-0.4	β-blocker agent
Stevioside	57,817–89–7	C38H60O18	804.3780	-2.16	Sweetener
Sucralose	56,038–13–2	C12H19Cl3O8	396.0146	-0.47	Sweetener
Sulfadiazine	68–35–9	C10H10N4O2S	250.0524	0.39	Antibiotic
Sulfadimethoxine	122–11–2	C12H14N4O4S	310.0736	0.23	Antibiotic
Sulfamerazine	127–79–7	C11H12N4O2S	264.0681	0.52	Antibiotic
Sulfamethazine	57–68–1	C12H14N4O2S	278.0837	0.65	Antibiotic
Sulfamethizole	144–82–1	C9H10N4O2S2	270.0245	0.21	Antibiotic
Sulfamethoxazole	723–46–6	C10H11N3O3S	253.0521	0.79	Antibiotic
Sulfapyridine	144–83–2	C11H11N3O2S	249.0572	1.01	Antibiotic
Sulfathiazole	72–14–0	C9H9N3O2S2	255.0136	0.98	Antibiotic
Sulisobenzone (BP4)	4065–45–6	C14H12O6S	308.0355	3.52	UV filter
Tramadol	3715–90–0	C16H25NO2	263.1885	2.45	Analgesic
Trimethoprim	738–70–5	C14H18N4O3	290.1379	1.28	Antibiotic
Valsartan	137,862–53–4	C24H29N5O3	435.2271	4.59	Antihypertensive
Valsartan acid	164,265–78–5	C14H10N4O2	266.0804	2.56	Antihypertensive TP / Metabolite
Venlafaxine	93,413–69–5	C17H27NO2	277.2042	2.74	Antidepressant
Verapamil	52–53–9	C27H38N2O4	454.2832	5.04	Calcium channel blocking agent
Warfarin	81–81–2	C19H16O4	308.1049	2.74	Anticoagulant
Zonisamide	68,291–97–4	C8H8N2O3S	212.0256	0.11	Anticonvulsant

⁎ Predicted values obtained from Chemaxon (www.chemaxon.com).

### Sampling and sample pre-treatment

Surface water samples were collected from six different municipalities from Osona, a region in Catalonia, Spain. These municipalities were chosen as they have their own WWTP and a direct discharge into their respective municipality stream. For method development and validation, samples were taken from upstream points, approximately 100 m before the WWTP discharge point to avoid possible direct contamination from the discharge points and were collected in 1-L amber PET bottles, which were previously rinsed with sample water on site. The bottles were placed in a refrigerator at 4 °C under dark conditions and transported to the laboratory. Once in the laboratory, samples were immediately filtered with 0.7-µm glass microfiber filter GF/F from Whatman (UK). An aliquot was stored in 0.5 L flasks to be extracted directly or stored in the dark at -20 °C. The remaining portion was blended with all upstream points to generate a representative pool of contaminant-free surface water from the six different streams to be used for method validation and the corresponding matrix-matched curve. In Table 2 some basic water parameters are shown (pH and electrical conductivity (EC)) for each of the studied municipalities and the sample pool made.

**Table 2:** Sampled upstream points from each municipality and sample pool water parameters.

**Municipalities studied (Osona, Spain)**	**pH**	**EC (μS cm^-1^)**	**Coordinates**
Alpens	8.06	520	42°06′56.5″N 2°05′53.0″E
Folgueroles	6.81	617	41°56′39.0″N 2°18′34.0″E
Santa Eulàlia de Riuprimer	7.98	940	41°54′44.5″N 2°11′35.2″E
Taradell	7.66	572	41°52′52.0″N 2°16′33.9″E
Vidrà	7.6	501	42°07′13.9″N 2°18′50.0″E
Viladrau	7.72	170	41°50′51.6″N 2°22′21.1″E
Sample Pool	7.63	514	-

### Extraction procedure

Aliquots of 500 mL of surface water pool were extracted using a solid phase extraction. A homemade multi-sorbent SPE cartridge, previously developed by [8], was used for the extraction. This cartridge consisted of two layers, an upper layer containing 200 mg of Oasis HLB sorbent and a bottom layer containing a mixture of 150 mg of Bond Elut PPL, 100 mg of WAX and 100 mg WCX. Activation and conditioning of the cartridges were performed by passing 2 x 5 mL of MeOH followed by 2 x 5 mL of HPLC water. Before the sample loading, a mix of 80 isotopically labelled compounds was added to every sample. Then the samples were loaded on the previously activated cartridges at one drop every two seconds approximately for the maximum retention of the compounds. After the samples were loaded, cartridges were then dried passing a slight air current through the cartridges. Cartridges were then eluted with 2 x 3 mL 5% NH_3_ in MeOH:EtOAc (50:50 v/v), followed by 2 x 3 mL of 2% formic acid in MeOH:EtOAc (50:50 v/v) and finally 3 mL of MeOH:EtOAc (50:50 v/v). The eluate (approximately 15 mL) was then evaporated to dryness under a gentle nitrogen stream and then reconstituted in 500 μL of H_2_O:MeOH (95:5 v/v). A final concentration factor of 1000 was obtained.

### Instrument analysis

The targeted contaminants of emerging concern were separated using ultra high-performance liquid chromatography in a Waters Acquity HSS T3 (C18) column (100 × 2.1 mm i.d., 1.8 µm particle size) thermostated at 40 °C using a Waters ACQUITY UHPLC system (Waters, Milford, MA). For the detection, a Q-Exactive Orbitrap mass spectrometer (Thermo-Fisher Scientific, Germany) equipped with heated electrospray ionization (HESI) was used. The tuning methods and parameters used for the MS acquisition were optimized in the following conditions; spray voltage was set to 3.5 kV (positive) and 2.5 kV (negative), S-Lens RF level, 60; capillary temperature, 350 °C; auxiliary gas temperature, 300 °C. Nitrogen was used as sheath gas flow rate 40; auxiliary gas flow rate 10 and sweep gas flow rate 2. For the positive electrospray ionization (ESI+), the mobile phases used were ACN and 5 mM AcNH_4_ + 0.1% formic acid in water while for the negative electrospray ionization (ESI-) mode ACN and 2 mM NH_4_F in water were used. The gradient profile was the same for both ionizations as shown in Table 3. The injection volume was set to 10 µL.

**Table 3:** Gradient profile, time, percentage of mobile phases and flow rate.

**Time (min)**	**Mobile** **phase A (%)**	**Mobile phase B (%)**	**Flow (min/mL)**
0.0	5.0	95.0	0.2
0.3	5.0	95.0	0.2
10.0	30.0	70.0	0.2
13.3	65.0	35.0	0.2
15.5	100.0	0.0	0.2
17.3	100.0	0.0	0.2
17.7	5.0	95.0	0.2
19.0	5.0	95.0	0.2

As for the mass spectrometer, a full-scan experiment followed by a targeted data-independent acquisition (targeted-DIA) mode were used with a specific predefined inclusion list which contained the exact mass of the selected precursor ion, optimized retention time windows and optimized collision energies in order to obtain high quality MS2 product ions (Table SM 2 and 3). For the retention time optimization, a mix of the analytes at 10 μg l^-1^ was initially evaluated with the specific selected column by a full-scan MS with a targeted data-dependent MS2 (dd-MS2) acquisition mode in order to obtain the compound retention times and study possible co-elutions. Then, the predefined inclusion list was compiled. The MS parameters for the selected method were optimized and described as follows: full MS (70,000 K resolution, AGC target 3x10^6^, IT 150 ms, scan range 90–1000 *m/z*) followed by a DIA experiment to generate MS2 spectra (17,500 resolution, AGC 2x10^5^, IT auto, Loop count 1, Top N 10, isolation window 1.5 *m/z*, and NCE 30 eV). As mentioned above, the inclusion list was included with the exact mass to generate as many micro-scanning events as precursor ions filtered. In this way, greater sensitivity and selectivity were achieved by this method. Further information on specific details is included in the supplementary material (Table SM 1) or elsewhere [4].

The guidance SANTE 11,312/2021 [9] was followed, and two ions were used for high-confidence peak identification criteria. For quantification, the presence of the precursor ion in the extracted chromatogram (signal-to-noise ratio >3) with a mass accuracy within 5 ppm and matched retention time within 0.1 min compared to the reference standard was applied. For further confirmation, the presence of at least one product ion in the MS2 spectrum with a mass error below 5 ppm was expected. Only the adduct ion obtained from the full-scan spectrum was used for the isotopically labelled compounds.

### Method validation

Surface water pool samples were used for the method validation, and aliquots of 500 mL were spiked with the mix of the 116 selected targeted compounds. The method was validated, in triplicate, at three different concentration levels: 5, 50, and 500 μg l^-1^ in the extract, which would correspond to final sample concentrations of 5, 50, 500 ng l^-1^ respectively, after applying the method concentration factor of 1000. These concentrations were chosen in order to cover common environmental concentrations. The method was evaluated in terms of accuracy (recovery rates), matrix effect (ME), precision (% RSD), linearity (R^2^), method detection limits (MDL), method quantification limits (MQL) according to [10], for all three validated concentrations.

In order to evaluate the accuracy of the method developed, recovery rates (RR) were calculated, for both intra and inter day precision. The recovery rates were calculated by comparing the peak area of the pool samples spiked before and after the method extraction. A blank sample without any spiked compounds was subtracted in each case (Eq. (1))

(1) $$RR\;\left(\%\right)=\frac{Area_{pre-spiked}-Area_{blank}}{Area_{post-spiked}-Area_{blank}}\;x\;100$$

**Fig. 1:**
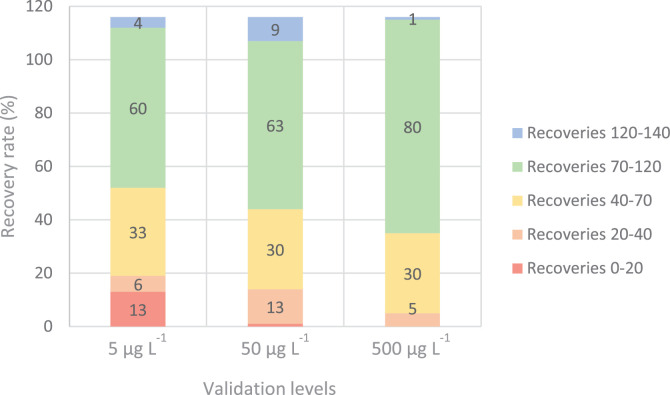
Recovery rates for the three validated concentrations in surface water matrices.

The recovery rates obtained for the present method are shown in Fig. 1. The recovery rates were generally good for most of the compounds ranging from 70 to 120% with the exception of some compounds such as cyclamate, irgasan, losartan, lidocaine or paroxetine which presented recovery values of 26, 20, 19, 38 and 19% respectively at the lowest level of validation.  Nevertheless, for the high validation level (500 μg l^-1^ in the extract) more than 80 compounds were recovered with recovery values higher than 70%. At the same time, those compounds with low recovery rates at the low validation level, are usually found at high concentrations in the environment [11,12] and with the use of isotopically labelled compounds these can be quantified and thus those compounds were included in the method anyway.

Matrix effect was also studied to relate either signal suppression or enhancement and was calculated for the selected compounds and for the three validation levels. Eq. (2) was applied where the area from a spiked matrix sample (post-spiked) was compared with the area given in a spiked solvent sample at the same given concentration. The area from a blank matrix (non-spiked) was subtracted.

(2) $$\mathrm{ME}\left(\%\right)=[\left[\left(Area_{matrix}-Area_{blank}\right)/\left(Area_{solvent}\right)\right]\;...\;1]\times 100$$

**Fig. 2:**
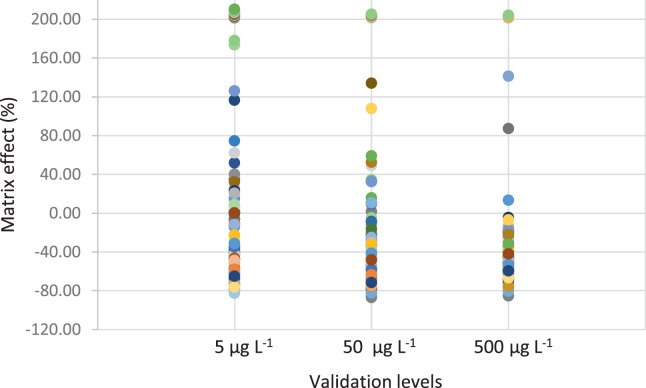
Matrix effect for the three validated concentrations in surface water matrices.

The matrix effects for the validated compounds for each validation level are shown in Fig. 2. Mainly ion suppression was observed in all three cases, and as the concentration increased, a slight increase or change from rather matrix enhancement to suppression was observed. There were some exceptional cases where an important enhancement was observed for hydroxychloroquine, azithromycin or carazolol.

Precision was evaluated by both intra and inter-day relative standard deviation (RSD), shown in Table 4, to study the repeatability and reproducibility. Good results were obtained for the studied method with intra-day RSD values from 0.43 to 20.63% and inter-day values from 0.06 to 19.31%, with the exception of crotamiton and fluticasone, which gave slightly higher RSDs.  MLODs and MLOQs were estimated by the standard deviation of the intercept divided by the slope, multiplied by three and ten respectively. Linearity range, MLODs and MLOQs values are given in concentrations found in final extract. The linearity interval of the matrix-matched calibration curve covered a wide range of concentrations from 0.05 to 1000 μg l^-1^ with R^2^ >0.99 for the majority of the compounds. The method also proved to be highly sensitive with MLODs in the range of 0.01–1.89 μg l^-1^ and MLOQs ranging from 0.05 to 6.31 μg l^-1^.

**Table 4:** Relative standard deviation, method limits of detection and quantification, and linearity (R*^2^*) for each compound.

**Compound name**	**Intraday RSD (%)**			**Interday RSD (%)**			**MLOD (ng mL^-1^)**	**MLOQ (ng mL^-1^)**	**R^2^**
	**5 ppb**	**50 ppb**	**500 ppb**	**5 ppb**	**50 ppb**	**500 ppb**			
1,7 Dimethylxanthine	–	9.70	5.72	–	9.91	13.41	0.25	0.84	0.9975
17-α Ethynilestradiol	18.57	18.99	12.35	8.11	3.24	1.91	0.09	0.30	0.9997
1H-Benzotriazole	1.46	3.16	7.40	5.99	3.22	1.22	0.12	0.39	0.9990
2.4-Dihydroxybenzophenone (BP1)	7.73	16.45	4.83	1.06	1.44	1.44	0.52	1.75	0.9918
2.2′.4.4′-Tetrahydroxybenzophenone (BP2)	13.45	17.70	5.49	2.42	1.19	1.13	0.19	0.63	0.9994
4-Hydroxidiclofenac	20.63	2.45	15.06	10.27	1.52	2.02	0.97	3.24	0.9999
4-Nitro Sulfamethoxazole	4.41	9.54	5.03	3.93	1.41	0.68	0.20	0.68	0.9985
5-Desamino-5 oxo 2,5 Lamotrigine	2.54	6.99	7.35	3.45	1.63	1.19	0.23	0.77	0.9929
5-methyl-1 H Benzotriazole	1.96	4.84	7.52	8.51	1.02	0.29	0.26	0.88	0.9968
5-nitro-Diclofenac	9.33	17.91	11.24	3.11	1.85	1.32	0.25	0.82	0.9981
6a-Methylprednisolone	18.91	20.23	17.23	13.32	3.36	0.91	0.41	1.36	0.9949
Acesulfame	11.21	17.47	8.93	12.65	5.62	3.24	0.50	1.68	0.9935
Acetaminophen	2.67	0.93	5.21	6.21	18.55	1.80	0.12	0.41	0.9996
Acridone	5.80	4.38	7.88	0.06	1.92	0.54	0.16	0.54	0.9992
Adamantan-1-amine (Amantadine)	3.76	6.20	7.00	2.31	2.32	1.38	0.13	0.43	0.9994
Alprazolam	3.01	6.86	4.53	0.96	2.87	3.43	0.42	1.40	0.9976
Atenolol	9.77	9.49	12.04	3.97	14.84	6.46	0.10	0.34	0.9999
Azithromycin	–	2.76	1.51	–	2.45	1.26	0.34	1.14	0.9959
Benzoylecgonine	1.18	6.22	6.12	0.57	0.81	1.78	0.32	1.08	0.9990
B-estradiol	15.96	12.68	11.20	16.83	0.47	2.90	0.37	1.24	0.9977
Bezafibrate	0.63	7.09	5.88	3.86	1.79	0.77	0.14	0.48	0.9996
Bisphenol-A	–	30.90	12.72	–	1.51	0.46	0.07	0.25	0.9992
Bromazepam	7.01	4.55	7.14	6.32	3.88	9.65	0.31	1.02	0.9999
Caffeine	–	7.01	4.05	–	12.88	7.78	0.14	0.46	0.9952
Carazolol	5.65	6.13	7.31	8.27	11.33	8.31	0.06	0.21	0.9979
Carbamazepine	1.26	4.64	6.41	3.54	1.49	0.27	0.11	0.35	0.9987
Carbamazepine-10,11-epoxide	3.13	1.56	3.31	2.56	1.72	0.08	0.19	0.64	0.9982
Carisoprodol	0.66	10.32	4.84	3.62	2.36	1.02	0.20	0.68	0.9983
Chloramphenicol	4.88	7.01	5.70	1.90	1.49	0.28	0.86	2.85	0.9936
Citalopram	5.65	7.67	3.63	1.36	1.42	0.79	0.11	0.37	0.9993
Clarithromycin	4.42	3.91	4.38	9.25	1.98	0.92	0.15	0.50	0.9975
Climbazole	6.44	17.87	8.42	1.28	0.85	0.98	0.06	0.20	0.9997
Clofibric Acid	3.37	8.59	4.83	10.58	3.33	0.43	0.32	1.08	0.9997
Cocaethylene	1.63	2.98	3.42	1.07	0.19	0.28	0.11	0.37	0.9996
Cocaine	2.71	3.36	6.62	2.70	0.81	1.09	0.16	0.54	0.9994
Codeine	8.58	7.10	10.01	12.92	3.68	1.39	0.13	0.43	0.9997
Cotinine	19.56	11.20	10.17	12.18	2.75	1.32	0.15	0.51	0.9991
Crotamiton	30.55	31.50	27.56	4.12	1.51	1.66	0.21	0.69	0.9961
Cyclamate	13.85	14.23	9.67	15.32	13.00	2.98	0.53	1.78	0.9991
Diazepam	3.39	9.70	5.72	1.09	1.08	0.79	0.12	0.40	0.9990
Diclofenac	18.83	15.39	18.62	19.31	2.03	1.03	1.51	5.05	0.9994
Diltiazem	10.21	4.39	4.06	0.93	1.41	1.44	0.14	0.47	0.9974
Estriol	2.07	8.31	4.40	18.24	2.11	3.13	0.14	0.46	0.9978
Estrone	6.99	12.43	8.22	5.51	2.15	2.78	0.32	1.05	0.9997
Ezetimibe	11.37	13.90	11.22	4.11	0.74	4.11	0.40	1.34	0.9915
Fipronil	18.46	13.65	13.33	1.17	2.31	2.40	0.34	1.13	0.9912
Fipronil desulfinyl	12.92	14.83	14.70	2.22	2.16	2.76	0.33	1.11	0.9907
Fipronil sulfide	13.63	16.59	13.10	1.37	1.22	4.07	0.44	1.48	0.9995
Fipronil sulfone	15.39	18.69	12.65	1.88	0.84	6.79	0.26	0.87	0.9976
Florfenicol	4.98	8.73	5.51	1.99	0.80	0.86	0.67	1.22	0.9918
Fluconazole	3.80	8.28	7.69	0.17	1.28	0.82	0.19	0.63	0.9998
Flumequine	0.90	4.91	8.36	1.40	1.10	0.66	0.07	0.25	0.9999
Fluticasone propionate	27.83	16.35	24.48	35.25	29.63	27.57	1.27	4.22	0.9998
Furazolidone	9.02	10.73	7.77	4.93	0.73	1.42	0.20	0.66	0.9932
Furosemide	9.69	9.96	3.60	13.42	1.19	0.86	0.18	0.60	0.9993
Gemfibrozil	7.31	12.16	6.55	18.69	17.19	16.17	0.25	0.84	0.9991
Hydrochlorothiazide	18.46	13.66	15.83	11.15	2.52	3.33	0.04	0.15	0.9997
Hydroxychloroquine	13.72	10.43	4.87	17.81	15.51	6.85	1.84	6.15	0.9966
Hyoscine (scopolamine)	3.45	6.21	7.68	2.81	1.82	0.54	0.11	0.37	0.9979
Ibuprofen	–	13.93	10.53	–	12.12	4.34	0.14	0.48	0.9988
Indomethacin	17.53	16.66	16.76	7.73	2.08	1.55	0.12	0.41	0.9997
Irbesartan	18.26	23.58	20.88	5.30	1.17	0.33	0.07	0.22	0.9996
Irgasan (Triclosan)	15.35	18.57	14.91	5.48	9.03	7.28	0.70	2.33	0.9978
Ketoprofen	8.86	7.50	6.98	10.92	0.70	1.27	0.36	1.18	0.9993
Lamotrigine	3.72	3.92	6.59	4.27	1.39	0.71	0.14	0.46	0.9976
Lamotrigine-N2-oxide	4.56	7.66	9.99	1.23	0.47	1.46	0.26	0.88	0.9915
Lidocaine	16.59	13.82	1.88	1.52	0.71	0.98	0.08	0.28	0.9998
Lincomycin	13.96	18.65	23.06	17.52	8.70	7.13	0.09	0.31	0.9963
Lorazepam	3.91	8.86	8.79	1.92	0.81	2.30	0.18	0.60	0.9987
Losartan	25.84	36.99	18.03	16.17	1.04	1.12	0.23	0.77	0.9965
Mefenamic Acid	15.79	17.58	12.63	5.70	2.13	1.19	0.46	1.53	0.9944
Meprobamate	6.70	5.04	0.52	2.09	0.78	0.89	0.75	2.50	0.9964
Methadone	3.36	12.36	3.68	1.16	0.57	0.38	0.02	0.07	0.9993
Metoprolol	1.96	6.95	5.65	1.14	0.06	0.23	0.06	0.20	0.9998
Metronidazole	5.75	4.12	8.38	4.27	1.73	3.04	0.02	0.08	0.9999
Midazolam	27.50	7.58	20.70	1.45	1.35	1.11	0.11	0.38	0.9936
Morphine	11.52	15.35	13.66	2.13	1.11	2.71	0.14	0.45	0.9936
N-acetyl sulfamethoxazole	2.69	8.43	7.86	5.55	1.35	0.53	0.16	0.52	0.9947
Nalidixic acid	0.47	4.65	9.51	2.10	1.13	0.11	0.12	0.41	0.9975
Naproxen	–	12.18	9.66	–	12.66	4.12	0.43	1.43	0.9973
Neotame	11.93	7.03	6.05	12.22	1.08	1.06	0.17	0.56	0.9962
N-Methyl lamotrigine	1.93	5.76	7.22	1.92	0.78	0.75	0.29	0.97	0.9932
O-desmethylvenlafaxine	5.72	4.45	2.49	1.27	0.45	1.25	0.19	0.63	0.9990
Oseltamivir	2.96	3.19	3.25	1.96	1.81	0.65	0.10	0.34	0.9997
Oxazepam	6.42	7.22	2.62	1.79	0.82	1.71	0.12	0.39	0.9998
Paroxetine	13.15	17.78	6.21	11.62	5.52	1.62	0.15	0.50	0.9994
Pentobarbital	4.38	4.57	1.31	1.53	1.14	1.34	0.16	0.55	0.9994
Phenytoin	3.53	8.81	6.36	1.40	1.17	1.67	0.27	0.90	0.9987
Primidone	–	9.60	11.60	–	7.33	1.60	1.89	6.31	0.9909
Propranolol	2.25	2.99	4.93	6.70	3.33	0.71	0.21	0.70	0.9984
Propyphenazone	1.38	11.66	1.34	2.27	1.47	0.48	0.18	0.60	0.9977
Rosuvastatin	2.43	9.24	6.10	3.99	0.28	0.47	0.20	0.66	0.9992
Saccharin	4.97	7.93	8.37	16.14	19.78	3.30	0.45	1.51	0.9993
Salicylic acid	6.81	17.00	5.82	12.27	16.50	7.62	0.75	2.50	0.9994
Sertraline	4.13	14.69	14.24	3.82	1.45	1.60	0.40	1.32	0.9951
Sitagliptin	16.37	6.38	2.58	9.34	0.69	0.37	0.21	0.70	0.9999
Sotalol	7.12	8.27	6.40	5.25	2.25	1.81	0.07	0.22	0.9990
Stevioside	–	8.31	8.31	–	5.68	1.25	0.35	1.18	0.9971
Sucralose	–	5.41	5.16	–	3.05	5.11	0.26	0.86	0.9974
Sulfadiazine	14.01	11.56	14.04	1.09	1.19	1.37	0.14	0.48	0.9999
Sulfadimethoxine	19.41	19.57	14.91	0.70	1.92	11.43	0.12	0.41	0.9995
Sulfamerazine	17.97	17.31	15.30	1.32	1.67	1.88	0.10	0.35	0.9992
Sulfamethazine	12.10	18.02	15.38	1.44	0.64	0.98	0.32	1.06	0.9972
Sulfamethizole	10.84	15.73	18.34	1.92	0.01	0.83	0.05	0.17	0.9999
Sulfamethoxazole	10.00	7.26	6.79	2.08	0.25	1.01	0.24	0.80	0.9914
Sulfapyridine	15.42	12.96	8.59	0.39	0.88	1.12	0.07	0.39	0.9991
Sulfathiazole	17.29	14.00	8.52	1.63	1.89	1.47	0.12	0.39	0.9988
Sulisobenzone (BP4)	8.27	10.55	5.02	3.02	0.35	1.06	0.10	0.34	0.9997
Tramadol	9.32	6.91	2.72	2.54	1.34	0.78	0.19	0.64	0.9907
Trimethoprim	1.67	3.36	6.72	0.89	0.76	1.07	0.14	0.47	0.9959
Valsartan	7.07	13.14	6.50	7.34	3.70	0.43	0.19	0.63	0.9998
Valsartan acid	22.81	5.10	2.81	18.09	1.71	0.88	0.61	2.05	0.9971
Venlafaxine	4.07	2.73	3.47	3.78	0.64	1.22	0.01	0.05	0.9999
Verapamil	9.33	16.19	10.66	1.66	0.72	0.72	0.11	0.36	0.9992
Warfarin	10.69	19.95	10.57	7.41	0.59	1.36	0.22	0.74	0.9955
Zonisamide	–	9.14	6.48	–	9.37	1.47	1.58	5.22	0.9993

Quality assurance and quality control were ensured by preparing procedural blanks using HPLC water and repeating the exposed method procedure. No peaks were observed for any of the target analytes. Quality controls were also prepared by spiking the same solvent ratio as the final extracts with standards and isotopically labelled compounds to reach a concentration of 50 μg l^-1^ in the vial, and these were injected and analysed along the sample sequence analysis to ensure reliable determination. To overcome matrix effects, isotopically labelled compounds were used in all samples as well as a matrix-matched calibration curve. For proper quantification, the matrix-matched calibration curve included 10 points with the 116 mix standards and 80 isotopically labelled compounds.

### Method applicability

**Fig. 3:**
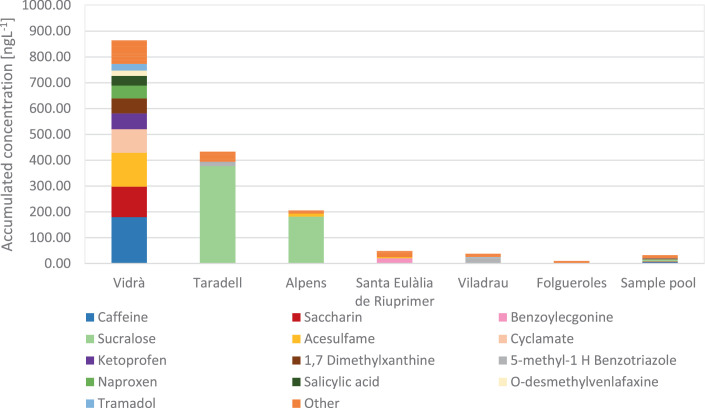
Total concentration of wastewater borne contaminants in the different Catalan municipalities and the surface water sample pool.

The WWTP discharge upstream samples of each municipality and the surface water pool used for the method validation and matrix-matched calibration curve (Table 2) were analysed as an example of method applicability and results are shown in Fig. 3. In general, very low levels of contamination were found. The most contaminated upstream was the Vidrà municipality with a total accumulated concentration of 864.2 ng L ^-1^, followed by the Taradell municipality with 433.7 ng L ^-1^.  In Fig. 3, those contaminants present with at least 20 ng L ^-1^ in one sample are shown. As expected, many of the selected contaminants were not detected (BP4, diclofenac, oxazepam, valsartan) as their presence is mainly expected in surface rivers after the wastewater treatment plant discharge. However, a total of 48 compounds of the 116 targeted compounds were detected in at least one of the samples. The highest concentrations found were mainly concerning those compounds used frequently in our day to day life such as sucralose (sweetener) with the highest concentration of 376.9 ng L ^-1^ found in Taradell followed by caffeine (stimulant), acesulfame or saccharin (sweeteners) with maximum concentrations of 179.4, 130.3 and 119.0 ng L ^-1^ respectively.

Overall, the method developed proved a very good performance for the quantification of a wide range of wastewater-borne pollutants with different polarities. Thus, it has a high applicability for monitoring and controlling the impact of wastewater-borne pollutants in surface water and to further explore possible other contamination sources other than the continuous WWTP discharges.

## CRediT author statement

**Olga Gómez-Navarro:** Methodology, Validation, Formal analysis, writing - Original Draft; Writing - Original Draft; **Francesc Labad:** sampling, laboratory work; **Diana P. Manjarrés:** sampling, laboratory work; **Sandra Pérez:** Conceptualization, Supervision, Project administration, Writing - Review & Editing, Funding acquisition; Nicola Montemurro*: Supervision, Methodology, Validation, Writing - Review & Editing

## Declaration of Competing Interests

The authors declare that they have no known competing financial interests or personal relationships that could have appeared to influence the work reported in this paper

## Data Availability

Data will be made available on request.

## Appendix Supplementary materials

Image, application 1
