# Supplementary material for: HRMS-Targeted-DIA methodology for quantification of wastewater-borne pollutants in surface water
Source: MethodsX. 2023 Feb 24;10:102093. doi: 10.1016/j.mex.2023.102093 (PMC10011426; doi:10.1016/j.mex.2023.102093)
Supplement: Supplementary file 1 [file mmc1.docx]

**Supplementary Material**

**Table SM 1. Target compound name, Cas number, Molecular formula, Isomeric SMILES and lnChlKey**

| **Compound name** | **Cas number** | **Molecular formula** | **Isomeric SMILES** | **InChIKey** |
| --- | --- | --- | --- | --- |
| 1,7 Dimethylxanthine | 611-59-6 | C7H8N4O2 | N1C=NC2=C1C(=O)N(C(=O)N2)C | QUNWUDVFRNGTCO-UHFFFAOYSA-N |
| 17-α Ethynilestradiol | 57-63-6 | C20H24O2 | C[C@]12CC[C@H]3[C@H]([C@@H]1CC[C@]2(C#C)O)CCC4=C3C=CC(=C4)O | BFPYWIDHMRZLRN-SLHNCBLASA-N |
| 1H-Benzotriazole | 95-14-7 | C6H5N3 | C1=CC2=NNN=C2C=C1 | QRUDEWIWKLJBPS-UHFFFAOYSA-N |
| 2.4-Dihydroxybenzophenone (BP1) | 131-56-6 | C13H10O3 | C1=CC=C(C=C1)C(=O)C2=C(C=C(C=C2)O)O | ZXDDPOHVAMWLBH-UHFFFAOYSA-N |
| 2.2'.4.4'-Tetrahydroxybenzophenone (BP2) | 131-55-5 | C13H10O5 | C1=CC(=C(C=C1O)O)C(=O)C2=C(C=C(C=C2)O)O | WXNRYSGJLQFHBR-UHFFFAOYSA-N |
| 4-Hydroxidiclofenac | 64118-84-9 | C14H11Cl2NO3 | C1=CC=C(C(=C1)CC(=O)O)NC2=C(C=C(C=C2Cl)O)Cl | KGVXVPRLBMWZLG-UHFFFAOYSA-N |
| 4-Nitro Sulfamethoxazole | 29699-89-6 | C10H9N3O5S | CC1=CC(=NO1)NS(=O)(=O)C2=CC=C(C=C2)[N+](=O)[O-] | XHJLDAHBWWMCRZ-UHFFFAOYSA-N |
| 5-Desamino-5 oxo 2,5 Lamotrigine | 252186-78-0 | C9H6Cl2N4O | C1=CC(=C(C(=C1)Cl)Cl)C2=NN=C(NC2=O)N | OVNGWOHWYFBGKF-UHFFFAOYSA-N |
| 5-methyl-1 H Benzotriazole | 136-85-6 | C7H7N3 | CC1=CC2=NNN=C2C=C1 | LRUDIIUSNGCQKF-UHFFFAOYSA-N |
| 5-nitro-Diclofenac | 174316-61-1 | C14H10Cl2N2O4 | ClC1=CC=CC(Cl)=C1NC2=CC=C([N+]([O-])=O)C=C2CC(O)=O | MRJVSFHDJGEONX-UHFFFAOYSA-N |
| 6a-Methylprednisolone | 83-43-2 | C22H30O5 | C[C@H]1C[C@H]2[C@@H]3CC[C@@]([C@]3(C[C@@H]([C@@H]2[C@@]4(C1=CC(=O)C=C4)C)O)C)(C(=O)CO)O | VHRSUDSXCMQTMA-PJHHCJLFSA-N |
| Acesulfame | 33665-90-6 | C4H5NO4S | CC1=CC(=O)NS(=O)(=O)O1 | YGCFIWIQZPHFLU-UHFFFAOYSA-N |
| Acetaminophen | 103-90-2 | C8H9NO2 | CC(=O)NC1=CC=C(C=C1)O | RZVAJINKPMORJF-UHFFFAOYSA-N |
| Acridone | 578-95-0 | C13H9NO | C1=CC=C2C(=C1)C(=O)C3=CC=CC=C3N2 | FZEYVTFCMJSGMP-UHFFFAOYSA-N |
| Adamantan-1-amine (Amantadine) | 768-94-5 | C10H17N | C1C2CC3CC1CC(C2)(C3)N | DKNWSYNQZKUICI-UHFFFAOYSA-N |
| Alprazolam | 28981-97-7 | C17H13ClN4 | CC1=NN=C2N1C3=C(C=C(C=C3)Cl)C(=NC2)C4=CC=CC=C4 | VREFGVBLTWBCJP-UHFFFAOYSA-N |
| Atenolol | 29122-68-7 | C14H22N2O3 | CC(C)NCC(COC1=CC=C(C=C1)CC(=O)N)O | METKIMKYRPQLGS-UHFFFAOYSA-N |
| Azithromycin | 83905-01-5 | C38H72N2O12 | CC[C@@H]1[C@@]([C@@H]([C@H](N(C[C@@H](C[C@@]([C@@H]([C@H]([C@@H]([C@H](C(=O)O1)C)O[C@H]2C[C@@]([C@H]([C@@H](O2)C)O)(C)OC)C)O[C@H]3[C@@H]([C@H](C[C@H](O3)C)N(C)C)O)(C)O)C)C)C)O)(C)O | MQTOSJVFKKJCRP-BICOPXKESA-N |
| Benzoylecgonine | 519-09-5 | C16H19NO4 | CN1[C@H]2CC[C@@H]1[C@H]([C@H](C2)OC(=O)C3=CC=CC=C3)C(=O)O | GVGYEFKIHJTNQZ-RFQIPJPRSA-N |
| B-estradiol | 50-28-2 | C18H24O2 | C[C@]12CC[C@H]3[C@H]([C@@H]1CC[C@@H]2O)CCC4=C3C=CC(=C4)O | VOXZDWNPVJITMN-ZBRFXRBCSA-N |
| Bezafibrate | 41859-67-0 | C19H20ClNO4 | CC(C)(C(=O)O)OC1=CC=C(C=C1)CCNC(=O)C2=CC=C(C=C2)Cl | IIBYAHWJQTYFKB-UHFFFAOYSA-N |
| Bisphenol-A | 80-05-7 | C15H16O2 | CC(C)(C1=CC=C(C=C1)O)C2=CC=C(C=C2)O | IISBACLAFKSPIT-UHFFFAOYSA-N |
| Bromazepam | 1812-30-2 | C14H10BrN3O | C1C(=O)NC2=C(C=C(C=C2)Br)C(=N1)C3=CC=CC=N3 | VMIYHDSEFNYJSL-UHFFFAOYSA-N |
| Caffeine | 58-08-2 | C8H10N4O2 | CN1C=NC2=C1C(=O)N(C(=O)N2C)C | RYYVLZVUVIJVGH-UHFFFAOYSA-N |
| Carazolol | 57775-29-8 | C18H22N2O2 | CC(C)NCC(COC1=CC=CC2=C1C3=CC=CC=C3N2)O | BQXQGZPYHWWCEB-UHFFFAOYSA-N |
| Carbamazepine | 298-46-4 | C15H12N2O | C1=CC=C2C(=C1)C=CC3=CC=CC=C3N2C(=O)N | FFGPTBGBLSHEPO-UHFFFAOYSA-N |
| Carbamazepine-10,11-epoxide | 36507-30-9 | C15H12N2O2 | C1=CC=C2C(=C1)C3C(O3)C4=CC=CC=C4N2C(=O)N | ZRWWEEVEIOGMMT-UHFFFAOYSA-N |
| Carisoprodol | 78-44-4 | C12H24N2O4 | CCCC(C)(COC(=O)N)COC(=O)NC(C)C | OFZCIYFFPZCNJE-UHFFFAOYSA-N |
| Chloramphenicol | 56-75-7 | C11H12Cl2N2O5 | C1=CC(=CC=C1[C@H]([C@@H](CO)NC(=O)C(Cl)Cl)O)[N+](=O)[O-] | WIIZWVCIJKGZOK-RKDXNWHRSA-N |
| Citalopram | 59729-33-8 | C20H21FN2O | CN(C)CCCC1(C2=C(CO1)C=C(C=C2)C#N)C3=CC=C(C=C3)F | WSEQXVZVJXJVFP-UHFFFAOYSA-N |
| Clarithromycin | 81103-11-9 | C38H69NO13 | CC[C@@H]1[C@@]([C@@H]([C@H](C(=O)[C@@H](C[C@@]([C@@H]([C@H]([C@@H]([C@H](C(=O)O1)C)O[C@H]2C[C@@]([C@H]([C@@H](O2)C)O)(C)OC)C)O[C@H]3[C@@H]([C@H](C[C@H](O3)C)N(C)C)O)(C)OC)C)C)O)(C)O | AGOYDEPGAOXOCK-KCBOHYOISA-N |
| Climbazole | 38083-17-9 | C15H17ClN2O2 | CC(C)(C)C(=O)C(N1C=CN=C1)OC2=CC=C(C=C2)Cl | OWEGWHBOCFMBLP-UHFFFAOYSA-N |
| Clofibric Acid | 882-09-7 | C10H11ClO3 | CC(C)(C(=O)O)OC1=CC=C(C=C1)Cl | TXCGAZHTZHNUAI-UHFFFAOYSA-N |
| Cocaethylene | 529-38-4 | C18H23NO4 | CCOC(=O)[C@@H]1[C@H]2CC[C@H](N2C)C[C@@H]1OC(=O)C3=CC=CC=C3 | NMPOSNRHZIWLLL-XUWVNRHRSA-N |
| Cocaine | 50-36-2 | C17H21NO4 | CN1[C@H]2CC[C@@H]1[C@H]([C@H](C2)OC(=O)C3=CC=CC=C3)C(=O)OC | ZPUCINDJVBIVPJ-LJISPDSOSA-N |
| Codeine | 76-57-3 | C18H21NO3 | CN1CC[C@]23[C@@H]4[C@H]1CC5=C2C(=C(C=C5)OC)O[C@H]3[C@H](C=C4)O | OROGSEYTTFOCAN-DNJOTXNNSA-N |
| Cotinine | 486-56-6 | C10H12N2O | CN1[C@@H](CCC1=O)C2=CN=CC=C2 | UIKROCXWUNQSPJ-VIFPVBQESA-N |
| Crotamiton | 483-63-6 | C13H17NO | CCN(C1=CC=CC=C1C)C(=O)/C=C/C | DNTGGZPQPQTDQF-XBXARRHUSA-N |
| Cyclamate | 100-88-9 | C6H13NO3S | C1CCC(CC1)NS(=O)(=O)O | HCAJEUSONLESMK-UHFFFAOYSA-N |
| Diazepam | 439-14-5 | C16H13ClN2O | CN1C(=O)CN=C(C2=C1C=CC(=C2)Cl)C3=CC=CC=C3 | AAOVKJBEBIDNHE-UHFFFAOYSA-N |
| Diclofenac | 15307-86-5 | C14H11Cl2NO2 | C1=CC=C(C(=C1)CC(=O)O)NC2=C(C=CC=C2Cl)Cl | DCOPUUMXTXDBNB-UHFFFAOYSA-N |
| Diltiazem | 42399-41-7 | C22H26N204S | CC(=O)O[C@@H]1[C@@H](SC2=CC=CC=C2N(C1=O)CCN(C)C)C3=CC=C(C=C3)OC | HSUGRBWQSSZJOP-RTWAWAEBSA-N |
| Estriol | 50-27-1 | C18H24O3 | C[C@]12CC[C@H]3[C@H]([C@@H]1C[C@H]([C@@H]2O)O)CCC4=C3C=CC(=C4)O | PROQIPRRNZUXQM-ZXXIGWHRSA-N |
| Estrone | 53-16-7 | C18H22O2 | C[C@]12CC[C@H]3[C@H]([C@@H]1CCC2=O)CCC4=C3C=CC(=C4)O | DNXHEGUUPJUMQT-CBZIJGRNSA-N |
| Ezetimibe | 163222-33-1 | C24H21F2NO3 | C1=CC(=CC=C1[C@@H]2[C@H](C(=O)N2C3=CC=C(C=C3)F)CC[C@@H](C4=CC=C(C=C4)F)O)O | OLNTVTPDXPETLC-XPWALMASSA-N |
| Fipronil | 120068-37-3 | C12H4Cl2F6N4OS | C1=C(C=C(C(=C1Cl)N2C(=C(C(=N2)C#N)S(=O)C(F)(F)F)N)Cl)C(F)(F)F | ZOCSXAVNDGMNBV-UHFFFAOYSA-N |
| Fipronil desulfinyl | 205650-65-3 | C12H4Cl2F6N4 | C1=C(C=C(C(=C1Cl)N2C(=C(C(=N2)C#N)C(F)(F)F)N)Cl)C(F)(F)F | JWKXVHLIRTVXLD-UHFFFAOYSA-N |
| Fipronil sulfide | 120067-83-6 | C12H4Cl2F6N4S | C1=C(C=C(C(=C1Cl)N2C(=C(C(=N2)C#N)SC(F)(F)F)N)Cl)C(F)(F)F | FQXWEKADCSXYOC-UHFFFAOYSA-N |
| Fipronil sulfone | 120068-36-2 | C12H4Cl2F6N4O2S | C1=C(C=C(C(=C1Cl)N2C(=C(C(=N2)C#N)S(=O)(=O)C(F)(F)F)N)Cl)C(F)(F)F | LGHZJDKSVUTELU-UHFFFAOYSA-N |
| Florfenicol | 73231-34-2 | C12H14Cl2FNO4S | CS(=O)(=O)C1=CC=C(C=C1)[C@H]([C@@H](CF)NC(=O)C(Cl)Cl)O | AYIRNRDRBQJXIF-NXEZZACHSA-N |
| Fluconazole | 86386-73-4 | C13H12F2N6O | C1=CC(=C(C=C1F)F)C(CN2C=NC=N2)(CN3C=NC=N3)O | RFHAOTPXVQNOHP-UHFFFAOYSA-N |
| Flumequine | 42835-25-6 | C14H12FNO3 | CC1CCC2=C3N1C=C(C(=O)C3=CC(=C2)F)C(=O)O | DPSPPJIUMHPXMA-UHFFFAOYSA-N |
| Fluticasone propionate | 80474-14-2 | C25H31F3O5S | CCC(=O)O[C@@]1([C@@H](C[C@@H]2[C@@]1(C[C@@H]([C@]3([C@H]2C[C@@H](C4=CC(=O)C=C[C@@]43C)F)F)O)C)C)C(=O)SCF | WMWTYOKRWGGJOA-CENSZEJFSA-N |
| Furazolidone | 67-45-8 | C8H7N3O5 | C1COC(=O)N1/N=C/C2=CC=C(O2)[N+](=O)[O-] | PLHJDBGFXBMTGZ-WEVVVXLNSA-N |
| Furosemide | 54-31-9 | C12H11ClN2O5S | C1=COC(=C1)CNC2=CC(=C(C=C2C(=O)O)S(=O)(=O)N)Cl | ZZUFCTLCJUWOSV-UHFFFAOYSA-N |
| Gemfibrozil | 25812-30-0 | C15H22O3 | CC1=CC(=C(C=C1)C)OCCCC(C)(C)C(=O)O | HEMJJKBWTPKOJG-UHFFFAOYSA-N |
| Hydrochlorothiazide | 58-93-5 | C7H8ClN3O4S2 | C1NC2=CC(=C(C=C2S(=O)(=O)N1)S(=O)(=O)N)Cl | JZUFKLXOESDKRF-UHFFFAOYSA-N |
| Hydroxychloroquine | 118-42-3 | C18H26ClN3O | CCN(CCCC(C)NC1=C2C=CC(=CC2=NC=C1)Cl)CCO | XXSMGPRMXLTPCZ-UHFFFAOYSA-N |
| Hyoscine (scopolamine) | 51-34-3 | C17H21NO4 | CN1[C@@H]2CC(C[C@H]1[C@H]3[C@@H]2O3)OC(=O)[C@H](CO)C4=CC=CC=C4 | STECJAGHUSJQJN-USLFZFAMSA-N |
| Ibuprofen | 15687-27-1 | C13H18O2 | CC(C)CC1=CC=C(C=C1)C(C)C(=O)O | HEFNNWSXXWATRW-UHFFFAOYSA-N |
| Indomethacin | 53-86-1 | C19H16ClNO4 | CC1=C(C2=C(N1C(=O)C3=CC=C(C=C3)Cl)C=CC(=C2)OC)CC(=O)O | CGIGDMFJXJATDK-UHFFFAOYSA-N |
| Irbesartan | 138402-11-6 | C25H28N6O | CCCCC1=NC2(CCCC2)C(=O)N1CC3=CC=C(C=C3)C4=CC=CC=C4C5=NNN=N5 | YOSHYTLCDANDAN-UHFFFAOYSA-N |
| Irgasan (Triclosan) | 3380-34-5 | C12H7Cl3O2 | C1=CC(=C(C=C1Cl)O)OC2=C(C=C(C=C2)Cl)Cl | XEFQLINVKFYRCS-UHFFFAOYSA-N |
| Ketoprofen | 22071-15-4 | C16H14O3 | CC(C1=CC(=CC=C1)C(=O)C2=CC=CC=C2)C(=O)O | DKYWVDODHFEZIM-UHFFFAOYSA-N |
| Lamotrigine | 84057-84-1 | C9H7Cl2N5 | C1=CC(=C(C(=C1)Cl)Cl)C2=C(N=C(N=N2)N)N | PYZRQGJRPPTADH-UHFFFAOYSA-N |
| Lamotrigine-N2-oxide | 136565-76-9 | C9H7Cl2N5O | C1=CC(=C(C(=C1)Cl)Cl)C2=NN(C(=N)N=C2N)O | YGGNPWUXPLVPLK-UHFFFAOYSA-N |
| Lidocaine | 137-58-6 | C14H22N2O | CCN(CC)CC(=O)NC1=C(C=CC=C1C)C | NNJVILVZKWQKPM-UHFFFAOYSA-N |
| Lincomycin | 154-21-2 | C18H34N2O6S | CCC[C@@H]1C[C@H](N(C1)C)C(=O)N[C@@H]([C@@H]2[C@@H]([C@@H]([C@H]([C@H](O2)SC)O)O)O)[C@@H](C)O | OJMMVQQUTAEWLP-KIDUDLJLSA-N |
| Lorazepam | 846-49-1 | C15H10Cl2N2O2 | C1=CC=C(C(=C1)C2=NC(C(=O)NC3=C2C=C(C=C3)Cl)O)Cl | DIWRORZWFLOCLC-UHFFFAOYSA-N |
| Losartan | 114798-26-4 | C22H23ClN6O | CCCCC1=NC(=C(N1CC2=CC=C(C=C2)C3=CC=CC=C3C4=NNN=N4)CO)Cl | PSIFNNKUMBGKDQ-UHFFFAOYSA-N |
| Mefenamic Acid | 61-68-7 | C15H15NO2 | CC1=C(C(=CC=C1)NC2=CC=CC=C2C(=O)O)C | HYYBABOKPJLUIN-UHFFFAOYSA-N |
| Meprobamate | 57-53-4 | C9H18N2O4 | CCCC(C)(COC(=O)N)COC(=O)N | NPPQSCRMBWNHMW-UHFFFAOYSA-N |
| Methadone | 76-99-3 | C21H27NO | CCC(=O)C(CC(C)N(C)C)(C1=CC=CC=C1)C2=CC=CC=C2 | USSIQXCVUWKGNF-UHFFFAOYSA-N |
| Metoprolol | 37350-58-6 | C6H9N3O3 | CC(C)NCC(COC1=CC=C(C=C1)CCOC)O | IUBSYMUCCVWXPE-UHFFFAOYSA-N |
| Metronidazole | 443-48-1 | C15H25NO3 | CC1=NC=C(N1CCO)[N+](=O)[O-] | VAOCPAMSLUNLGC-UHFFFAOYSA-N |
| Midazolam | 59467-70-8 | C18H13ClFN3 | CC1=NC=C2N1C3=C(C=C(C=C3)Cl)C(=NC2)C4=CC=CC=C4F | DDLIGBOFAVUZHB-UHFFFAOYSA-N |
| Morphine | 57-27-2 | C17H19NO3 | CN1CC[C@]23[C@@H]4[C@H]1CC5=C2C(=C(C=C5)O)O[C@H]3[C@H](C=C4)O | BQJCRHHNABKAKU-KBQPJGBKSA-N |
| N-acetyl sulfamethoxazole | 21312-10-7 | C12H13N3O4S | CC1=CC(=NO1)NS(=O)(=O)C2=CC=C(C=C2)NC(=O)C | GXPIUNZCALHVBA-UHFFFAOYSA-N |
| Nalidixic acid | 389-08-2 | C12H12N2O3 | CCN1C=C(C(=O)C2=C1N=C(C=C2)C)C(=O)O | MHWLWQUZZRMNGJ-UHFFFAOYSA-N |
| Naproxen | 22204-53-1 | C14H14O3 | C[C@@H](C1=CC2=C(C=C1)C=C(C=C2)OC)C(=O)O | CMWTZPSULFXXJA-VIFPVBQESA-N |
| Neotame | 165450-17-9 | C20H30N2O5 | CC(C)(C)CCN[C@@H](CC(=O)O)C(=O)N[C@@H](CC1=CC=CC=C1)C(=O)OC | HLIAVLHNDJUHFG-HOTGVXAUSA-N |
| N-Methyl lamotrigine | 1373243-86-7 | C10H9Cl2N5 | CNC1=C(N=NC(=N1)N)C2=C(C(=CC=C2)Cl)Cl | WVJYMSFBMQWMAL-UHFFFAOYSA-N |
| O-desmethylvenlafaxine | 93413-62-8 | C16H25NO2 | CN(C)CC(C1=CC=C(C=C1)O)C2(CCCCC2)O | KYYIDSXMWOZKMP-UHFFFAOYSA-N |
| Oseltamivir | 196618-13-0 | C16H28N2O4 | CCC(CC)O[C@@H]1C=C(C[C@@H]([C@H]1NC(=O)C)N)C(=O)OCC | VSZGPKBBMSAYNT-RRFJBIMHSA-N |
| Oxazepam | 604-75-1 | C15H11ClN2O2 | C1=CC=C(C=C1)C2=NC(C(=O)NC3=C2C=C(C=C3)Cl)O | ADIMAYPTOBDMTL-UHFFFAOYSA-N |
| Paroxetine | 61869-08-7 | C19H20FNO3 | C1CNC[C@H]([C@@H]1C2=CC=C(C=C2)F)COC3=CC4=C(C=C3)OCO4 | AHOUBRCZNHFOSL-YOEHRIQHSA-N |
| Pentobarbital | 76-74-4 | C11H18N2O3 | CCCC(C)C1(C(=O)NC(=O)NC1=O)CC | WEXRUCMBJFQVBZ-UHFFFAOYSA-N |
| Phenytoin | 57-41-0 | C15H12N2O2 | C1=CC=C(C=C1)C2(C(=O)NC(=O)N2)C3=CC=CC=C3 | CXOFVDLJLONNDW-UHFFFAOYSA-N |
| Primidone | 125-33-7 | C12H14N2O2 | CCC1(C(=O)NCNC1=O)C2=CC=CC=C2 | DQMZLTXERSFNPB-UHFFFAOYSA-N |
| Propranolol | 525-66-6 | C16H21NO2 | CC(C)NCC(COC1=CC=CC2=CC=CC=C21)O | AQHHHDLHHXJYJD-UHFFFAOYSA-N |
| Propyphenazone | 479-92-5 | C14H18N2O | CC1=C(C(=O)N(N1C)C2=CC=CC=C2)C(C)C | PXWLVJLKJGVOKE-UHFFFAOYSA-N |
| Rosuvastatin | 287714-41-4 | C22H28FN3O6S | CC(C)C1=NC(=NC(=C1/C=C/[C@H](C[C@H](CC(=O)O)O)O)C2=CC=C(C=C2)F)N(C)S(=O)(=O)C | BPRHUIZQVSMCRT-VEUZHWNKSA-N |
| Saccharin | 81-07-2 | C7H5NO3S | C1=CC=C2C(=C1)C(=O)NS2(=O)=O | CVHZOJJKTDOEJC-UHFFFAOYSA-N |
| Salicylic acid | 69-72-7 | C7H6O3 | C1=CC=C(C(=C1)C(=O)O)O | YGSDEFSMJLZEOE-UHFFFAOYSA-N |
| Sertraline | 79617-96-2 | C17H17Cl2N | CN[C@H]1CC[C@H](C2=CC=CC=C12)C3=CC(=C(C=C3)Cl)Cl | VGKDLMBJGBXTGI-SJCJKPOMSA-N |
| Sitagliptin | 486460-32-6 | C16H15F6N5O | C1CN2C(=NN=C2C(F)(F)F)CN1C(=O)C[C@@H](CC3=CC(=C(C=C3F)F)F)N | MFFMDFFZMYYVKS-SECBINFHSA-N |
| Sotalol | 3930-20-9 | C12H20N2O3S | CC(C)NCC(C1=CC=C(C=C1)NS(=O)(=O)C)O | ZBMZVLHSJCTVON-UHFFFAOYSA-N |
| Stevioside | 57817-89-7 | C38H60O18 | C[C@@]12CCC[C@@]([C@H]1CC[C@]34[C@H]2CC[C@](C3)(C(=C)C4)O[C@H]5[C@@H]([C@H]([C@@H]([C@H](O5)CO)O)O)O[C@H]6[C@@H]([C@H]([C@@H]([C@H](O6)CO)O)O)O)(C)C(=O)O[C@H]7[C@@H]([C@H]([C@@H]([C@H](O7)CO)O)O)O | UEDUENGHJMELGK-HYDKPPNVSA-N |
| Sucralose | 56038-13-2 | C12H19Cl3O8 | ([C@@H]1[C@@H]([C@@H]([C@H]([C@H](O1)O[C@]2([C@H]([C@@H]([C@H](O2)CCl)O)O)CCl)O)O)Cl)O | BAQAVOSOZGMPRM-QBMZZYIRSA-N |
| Sulfadiazine | 68-35-9 | C10H10N4O2S | C1=CN=C(N=C1)NS(=O)(=O)C2=CC=C(C=C2)N | SEEPANYCNGTZFQ-UHFFFAOYSA-N |
| Sulfadimethoxine | 122-11-2 | C12H14N4O4S | COC1=NC(=NC(=C1)NS(=O)(=O)C2=CC=C(C=C2)N)OC | ZZORFUFYDOWNEF-UHFFFAOYSA-N |
| Sulfamerazine | 127-79-7 | C11H12N4O2S | CC1=NC(=NC=C1)NS(=O)(=O)C2=CC=C(C=C2)N | QPPBRPIAZZHUNT-UHFFFAOYSA-N |
| Sulfamethazine | 57-68-1 | C12H14N4O2S | CC1=CC(=NC(=N1)NS(=O)(=O)C2=CC=C(C=C2)N)C | ASWVTGNCAZCNNR-UHFFFAOYSA-N |
| Sulfamethizole | 144-82-1 | C9H10N4O2S2 | CC1=NN=C(S1)NS(=O)(=O)C2=CC=C(C=C2)N | VACCAVUAMIDAGB-UHFFFAOYSA-N |
| Sulfamethoxazole | 723-46-6 | C10H11N3O3S | CC1=CC(=NO1)NS(=O)(=O)C2=CC=C(C=C2)N | JLKIGFTWXXRPMT-UHFFFAOYSA-N |
| Sulfapyridine | 144-83-2 | C11H11N3O2S | C1=CC=NC(=C1)NS(=O)(=O)C2=CC=C(C=C2)N | GECHUMIMRBOMGK-UHFFFAOYSA-N |
| Sulfathiazole | 72-14-0 | C9H9N3O2S2 | C1=CC(=CC=C1N)S(=O)(=O)NC2=NC=CS2 | JNMRHUJNCSQMMB-UHFFFAOYSA-N |
| Sulisobenzone (BP4) | 4065-45-6 | C14H12O6S | COC1=C(C=C(C(=C1)O)C(=O)C2=CC=CC=C2)S(=O)(=O)O | CXVGEDCSTKKODG-UHFFFAOYSA-N |
| Tramadol | 3715-90-0 | C16H25NO2 | C1CCC2=C(C1)C=CC=C2NC3=NCCN3.Cl | RZOXEODOFNEZRS-UHFFFAOYSA-N |
| Trimethoprim | 738-70-5 | C14H18N4O3 | COC1=CC(=CC(=C1OC)OC)CC2=CN=C(N=C2N)N | IEDVJHCEMCRBQM-UHFFFAOYSA-N |
| Valsartan | 137862-53-4 | C24H29N5O3 | CCCCC(=O)N(CC1=CC=C(C=C1)C2=CC=CC=C2C3=NNN=N3)[C@@H](C(C)C)C(=O)O | ACWBQPMHZXGDFX-QFIPXVFZSA-N |
| Valsartan acid | 164265-78-5 | C14H10N4O2 | C1=CC=C(C(=C1)C2=CC=C(C=C2)C(=O)O)C3=NNN=N3 | USAWIVMZUYOXCF-UHFFFAOYSA-N |
| Venlafaxine | 93413-69-5 | C17H27NO2 | CN(C)CC(C1=CC=C(C=C1)OC)C2(CCCCC2)O | PNVNVHUZROJLTJ-UHFFFAOYSA-N |
| Verapamil | 52-53-9 | C27H38N2O4 | CC(C)C(CCCN(C)CCC1=CC(=C(C=C1)OC)OC)(C#N)C2=CC(=C(C=C2)OC)OC | SGTNSNPWRIOYBX-UHFFFAOYSA-N |
| Warfarin | 81-81-2 | C19H16O4 | CC(=O)CC(C1=CC=CC=C1)C2=C(C3=CC=CC=C3OC2=O)O | PJVWKTKQMONHTI-UHFFFAOYSA-N |
| Zonisamide | 68291-97-4 | C8H8N2O3S | C1=CC=C2C(=C1)C(=NO2)CS(=O)(=O)N | UBQNRHZMVUUOMG-UHFFFAOYSA-N |

**Table SM 2 MS detailed parameters**

| **Full MS parameters** |  |
| --- | --- |
| *Settings* |  |
| Chrom. Peak width (FWHM) | 12 s |
| Microscans | 1 |
| Resolution | 70k |
| AGC target | 3e6 |
| Maximum IT | 150 ms |
| Number of scan ranges | 1 |
| Scan range | 90 to 1000 m/z |
| Spectrum data type | Profile |
| **MS/MS parameters** |  |
| *Settings* | DIA |
| Microscans | 1 |
| Resolution | 17.5k |
| AGC target | 2e5 |
| Maximum IT | Auto |
| Loop count | 1 |
| MSX count | 1 |
| MSX isochronous ITs | On |
| Top N | - |
| Isolation window | 1.5 m/z |
| Isolation offset | 0.0 m/z |
| Scan range | - |
| Spectrum data type | Profile |
| (N) CE / stepped (N) CE | NCE: 30 |
| Minimum AGC target | - |
| Intensity threshold | - |
| Apex trigger | - |
| Exclude isotopes | - |
| Dynamic exclusion | - |

**Table SM 3 Targeted-DIA Inclusion list for positive ESI**

| **Polarity** | **Start (min)** | **End (min)** | **(N)CE** | **(N)CE type** | **Compound Name** | **Mass (m/z)** |
| --- | --- | --- | --- | --- | --- | --- |
| Positive | 4.5 | 5.5 | 28 | CE | 1,7 Dimethylxanthine | 181.072 |
| Positive | 6.5 | 7.5 | 20 | CE | 1H Benzotriazole | 120.0556 |
| Positive | 13.5 | 14.5 | 18 | CE | 4-Hydroxidiclofenac | 312.0189 |
| Positive | 9.7 | 10.7 | 25 | CE | 5-Desamino-5 oxo 2 Lamotrigine | 256.9992 |
| Positive | 9.25 | 10 | 25 | CE | 5-methyl-1 H Benzotriazole | 134.0713 |
| Positive | 12.6 | 13.6 | 22 | CE | 6a-Methylprednisolone | 375.2166 |
| Positive | 4 | 4.75 | 20 | CE | Acetaminophen | 152.0706 |
| Positive | 12 | 13 | 30 | NCE | Acridone | 196.0757 |
| Positive | 7 | 7.8 | 15 | CE | Adamantan-1-amine (Amantadine) | 152.1434 |
| Positive | 13 | 14.1 | 37 | CE | Alprazolam | 309.0902 |
| Positive | 4 | 5 | 25 | CE | Atenolol | 267.1703 |
| Positive | 10.2 | 11.2 | 44 | CE | Azithromycin | 749.5158 |
| Positive | 7 | 8.2 | 26 | CE | Benzoylecgonine | 290.1387 |
| Positive | 13.8 | 15 | 14 | CE | Bezafibrate | 362.1154 |
| Positive | 12 | 13.1 | 31 | CE | Bromazepam | 316.008 |
| Positive | 5.5 | 6.5 | 27 | CE | Caffeine | 195.0877 |
| Positive | 10.8 | 11.8 | 40 | NCE | Carazolol | 299.1754 |
| Positive | 12.8 | 13.8 | 23 | CE | Carbamazepine | 237.1022 |
| Positive | 13.2 | 14.2 | 20 | CE | Carisoprodol | 261.1809 |
| Positive | 11.3 | 12.4 | 20 | CE | Carbamazepine-10,11 epoxide | 253.0972 |
| Positive | 12.2 | 13.3 | 26 | CE | Citalopram | 325.1711 |
| Positive | 13.1 | 14.1 | 40 | CE | Clarithromycin | 748.4842 |
| Positive | 14 | 15 | 30 | CE | Climbazole | 293.1051 |
| Positive | 9.8 | 10.8 | 30 | CE | Cocaine | 304.1543 |
| Positive | 11.2 | 12.3 | 25 | CE | Cocaethylene | 318.1700 |
| Positive | 5 | 6.1 | 42 | CE | Codeine | 300.1594 |
| Positive | 2.5 | 3.4 | 28 | CE | Cotinine | 177.1022 |
| Positive | 14.2 | 15.2 | 24 | CE | Crotamiton | 204.1383 |
| Positive | 14.5 | 15.5 | 35 | CE | Diazepam | 285.0789 |
| Positive | 15 | 16 | 23 | CE | Diclofenac | 296.024 |
| Positive | 12.5 | 13.5 | 24 | CE | Diltiazem | 415.1686 |
| Positive | 8.7 | 9.7 | 20 | CE | Fluconazole | 307.1113 |
| Positive | 13 | 14 | 30 | CE | Flumequine | 262.0874 |
| Positive | 15.3 | 16.3 | 20 | CE | Fluticasone propionate | 501.1917 |
| Positive | 8.4 | 9.5 | 18 | CE | Furazolidone | 226.0459 |
| Positive | - | - | - | - | Hydroxychloroquine | 336.1837 |
| Positive | 10.2 | 11.4 | 30 | CE | Hyoscine (scopolamine) | 360.2169 |
| Positive | 13.8 | 14.8 | 25 | CE | Irbesartan | 429.2397 |
| Positive | 13.8 | 14.8 | 25 | CE | Ketoprofen | 255.1016 |
| Positive | 8 | 9.3 | 40 | CE | Lamotrigine | 256.0151 |
| Positive | 7.5 | 8.5 | 18 | CE | Lidocaine | 235.1805 |
| Positive | 5.5 | 6.7 | 24 | CE | Lincomycin | 407.221 |
| Positive | 8 | 9.1 | 19 | CE | Lamotrigine-N2-oxide | 272.0100 |
| Positive | 13.3 | 14.3 | 26 | CE | Lorazepam | 321.103 |
| Positive | 13.2 | 14.2 | 20 | CE | Losartan | 423.1695 |
| Positive | 15.5 | 16.5 | 21 | CE | Mefenamic acid | 242.1176 |
| Positive | 10.2 | 11.5 | 13 | CE | Meprobamate | 219.1339 |
| Positive | 13 | 14.2 | 18 | CE | Methadone | 310.2165 |
| Positive | 4.3 | 5.3 | 17 | CE | Metronidazole | 172.0717 |
| Positive | 8.7 | 9.7 | 21 | CE | Metoprolol | 268.1907 |
| Positive | 12.2 | 13.3 | 32 | CE | Midazolam | 326.0855 |
| Positive | 3 | 4 | 40 | CE | Morphine | 286.1438 |
| Positive | 10.5 | 11.6 | 30 | CE | N-acetyl Sulfamethoxazole | 296.07 |
| Positive | 12.8 | 13.8 | 32 | CE | Nalidixic acid | 233.0921 |
| Positive | 12.5 | 13.5 | 30 | CE | Neotame | 379.2228 |
| Positive | 9.6 | 10.7 | 40 | CE | N-Methyl lamotrigine | 270.0308 |
| Positive | 7.7 | 9 | 21 | CE | O-desmethylvenlafaxine | 264.1958 |
| Positive | 11.1 | 12.1 | 18 | CE | Oseltamivir | 313.2122 |
| Positive | 13 | 14 | 25 | CE | Oxazepam | 287.0582 |
| Positive | 12.6 | 13.6 | 33 | CE | Paroxetine | 330.15 |
| Positive | 8 | 9 | 19 | CE | Primidone | 219.1128 |
| Positive | 11.7 | 12.75 | 23 | CE | Propranolol | 260.1645 |
| Positive | 13.2 | 14.3 | 30 | CE | Propyphenazone | 231.1492 |
| Positive | 13.5 | 14.5 | 43 | CE | Rosuvastatin | 482.1756 |
| Positive | 13 | 14.25 | 20 | CE | Sertraline | 306.0811 |
| Positive | 10 | 11 | 24 | CE | Sitagliptin | 408.1254 |
| Positive | 4 | 5 | 24 | CE | Sotalol | 273.1267 |
| Positive | 5.2 | 6.2 | 20 | CE | Sulfadiazine | 251.0597 |
| Positive | 6.5 | 7.6 | 21 | CE | Sulfamerazine | 265.0754 |
| Positive | 7.6 | 9 | 22 | CE | Sulfamethazine | 279.091 |
| Positive | 7.5 | 8.6 | 16 | CE | Sulfamethizole | 271.0318 |
| Positive | 10.3 | 11.3 | 20 | CE | Sulfamethoxazole | 254.0594 |
| Positive | 11.8 | 12.9 | 35 | NCE | Sulfadimethoxine | 311.0809 |
| Positive | 6.2 | 7.4 | 22 | CE | Sulfapyridine | 250.0645 |
| Positive | 5.8 | 7 | 15 | CE | Sulfathiazole | 256.0209 |
| Positive | 8.9 | 10 | 15 | CE | Tramadol | 264.1958 |
| Positive | 6.5 | 8 | 29 | CE | Trimethoprim | 291.1452 |
| Positive | 14 | 15 | 17 | CE | Valsartan | 436.2343 |
| Positive | 11 | 12.5 | 20 | CE | Valsartan acid | 267.0877 |
| Positive | 11 | 12 | 27 | CE | Venlafaxine | 278.2115 |
| Positive | 13 | 14 | 27 | CE | Verapamil | 455.2904 |
| Positive | 14.5 | 15.5 | 18 | CE | Warfarin | 309.1121 |
| Positive | 9 | 10 | 12 | CE | Zonisamide | 213.0328 |

| **Polarity** | **Start (min)** | **End (min)** | **(N)CE** | **(N)CE type** | **Comment** | **Mass (m/z)** |
| --- | --- | --- | --- | --- | --- | --- |
| Negative | 14.3 | 15.3 | 55 | NCE | 17-a-Ethynilestradiol | 295.1703 |
| Negative | 8.6 | 11.6 | 35 | NCE | 4-Nitro sulfamethoxazole | 282.0190 |
| Negative | 2.4 | 3.4 | 20 | CE | Acesulfame | 161.9866 |
| Negative | 14 | 15 | 54 | CE | B-estradiol | 271.1703 |
| Negative | 13.7 | 14.7 | 31 | CE | Bisphenol-A | 227.1077 |
| Negative | 14 | 15 | 24 | CE | BP1 (2.4-Dihydroxybenzophenone) | 213.0557 |
| Negative | 12 | 13 | 19 | CE | BP2 (2.2'.4.4'-Tetrahydroxybenzophenone) | 245.0455 |
| Negative | 9.1 | 10.1 | 34 | CE | BP4 (Sulisobenzone) | 307.0281 |
| Negative | 11.3 | 12.3 | 13 | CE | Chloramphenicol | 321.0050 |
| Negative | 8.4 | 11.4 | 10 | CE | Clofibric Acid | 213.0324 |
| Negative | 4.1 | 5.1 | 34 | CE | Cyclamate | 178.0543 |
| Negative | 11.4 | 12.4 | 55 | NCE | Estriol | 287.1652 |
| Negative | 14.4 | 15.4 | 80 | NCE | Estrone | 269.1547 |
| Negative | 14.3 | 15.3 | 30 | NCE | Ezetimibe | 408.1416 |
| Negative | 15.5 | 16.5 | 20 | CE | Fipronil | 434.9314 |
| Negative | 15.7 | 16.7 | 10 | CE | Fipronil desulfinyl | 386.9644 |
| Negative | 15.9 | 16.9 | 25 | CE | Fipronil sulfide | 418.9365 |
| Negative | 15.8 | 16.8 | 25 | CE | Fipronil sulfone | 450.9263 |
| Negative | 10.3 | 11.3 | 14 | CE | Florfenicol | 355.9931 |
| Negative | 9.2 | 11.2 |  |  | Furosemide | 329.0004 |
| Negative | 15.2 | 16.2 | 14 | CE | Gemfibrozil | 249.1496 |
| Negative | 5.9 | 6.9 | 29 | CE | Hydrochlorothiazide | 295.9572 |
| Negative | 12.9 | 15.9 |  |  | Indomethacin | 356.0695 |
| Negative | 16 | 17 | 20 | CE | Irgasan (Triclosan) | 286.9439 |
| Negative | 10.5 | 14.5 | 13 | CE | Naproxen | 229.0870 |
| Negative | 12.5 | 14.5 |  |  | NO2-Diclofenac | 338.9939 |
| Negative | 12.9 | 13.9 | 60 | NCE | Pentobarbital | 225.1244 |
| Negative | 12.8 | 13.8 | 25 | CE | Phenytoin | 251.0826 |
| Negative | 3.3 | 4.3 | 36 | CE | Saccharin | 181.9917 |
| Negative | 4 | 5 | 16 | CE | Salicylic acid | 137.0244 |
| Negative | 12.1 | 13.1 | 20 | CE | Stevioside | 803.3706 |
| Negative | 7.1 | 8.1 | 22 | CE | Sucralose | 395.0072 |
| Negative | 5.9 | 6.9 | 20 | CE | Sulfathiazole | 254.0063 |

**Table SM 4. Targeted-DIA Inclusion list for negative ESI**

**Table SM 5. Targeted compound mass spectrometer details, ionization, retention time, precursor and product ion mass and isotopically labelled standard used.**

| **Compound Name** | **Ionization** | **Retention time (min)** | **Precursor (m/z)** | **Product ion (m/z)** | **Isotopically labelled standard** |
| --- | --- | --- | --- | --- | --- |
| 1,7 Dimethylxanthine | + | 4.9 | 181.0720 | 124.0506 | Caffeine 13C3 |
| 17-α Ethynilestradiol | - | 14.7 | 295.1704 | 267.1393 | 17b-Estradiol D5 |
| 1H-Benzotriazole | + | 7.4 | 120.0556 | 65.0389 | Benzotriazole D4 |
| 2.4-Dihydroxybenzophenone (BP1) | - | 14.54 | 213.0557 | 169.0663 | 17b-Estradiol D5 |
| 2.2'.4.4'-Tetrahydroxybenzophenone (BP2) | - | 12.5 | 245.0455 | 109.0298 | Ibersartan D6 |
| 4-Hydroxidiclofenac | + | 14.2 | 312.0189 | 231.0448 | Diclofenac 13C6 |
| 4-Nitro sulfamethoxazole | - | 10 | 282.0190 | 138.0201 | Sulfamethoxazole 13C6 |
| 5-Desamino-5 oxo 2,5 Lamotrigine | + | 10.5 | 256.9991 | 229.0041 | Lamotrigine 13C3 |
| 5-methyl-1 H Benzotriazole | + | 10 | 134.0713 | 79.0543 | Benzotriazole D4 |
| 5-nitro-Diclofenac | - | 13.1 | 338.9945 | 295.0031 | Ibersartan D6 |
| 6a-Methylprednisolone | + | 13.3 | 375.2166 | 357.2064 | Carbamazepine D10 |
| Acesulfame | - | 2.8 | 161.9867 | 82.0300 | Sulfamethoxazole 13C6 |
| Acetaminophen | + | 4.7 | 152.0706 | 110.0600 | Acetaminophen D4 |
| Acridone | + | 12.6 | 196.0757 | 167.0724 | Carbamazepine D10 |
| Adamantan-1-amine (Amantadine) | + | 7.6 | 152.1434 | 135.1169 | Benzotriazole D4 |
| Alprazolam | + | 13.8 | 309.0901 | 281.0714 | Alpralozam D5 |
| Atenolol | + | 4.5 | 267.1703 | 190.0862 | Sotalol D6 |
| Azithromycin | + | 10.9 | 749.5158 | 591.4216 | Carbamazepine D10 |
| Benzoylecgonine | + | 7.8 | 290.1387 | 168.1019 | Benzotriazole D4 |
| B-estradiol | - | 14.3 | 271.1704 | 145.0664 | 17b-Estradiol D5 |
| Bezafibrate | + | 14.4 | 362.1154 | 316.1101 | Bezafibrate D4 |
| Bisphenol-A | - | 14.2 | 227.1078 | 211.0769 | BisphenolA D8 |
| Sulisobenzone (BP4) | - | 9.5 | 307.0282 | 211.0405 | Furosemide D5 |
| Bromazepam | + | 12.7 | 316.0080 | 209.0948 | Bromazepam D4 |
| Caffeine | + | 6.4 | 195.0877 | 138.0663 | Caffeine 13C3 |
| Carazolol | + | 11.5 | 299.1754 | 116.1070 | Propranolol D7 |
| Carbamazepine | + | 13.3 | 237.1022 | 194.0964 | Carbamazepine D10 |
| Carbamazepine-10,11-epoxide | + | 12 | 253.0972 | 180.0810 | Carbamazepine D10 |
| Carisoprodol | + | 13.9 | 261.1809 | 62.0240 | Carisoprodol D7 |
| Chloramphenicol | - | 11.7 | 321.0051 | 152.0357 | Ibersartan D6 |
| Citalopram | + | 12.9 | 325.1711 | 109.0448 | Midazolam 13C6 |
| Clarithromycin | + | 13.8 | 748.4841 | 158.1178 | Carbamazepine D10 |
| Climbazole | + | 14.7 | 293.1051 | 69.0700 | Climbazole D4 |
| Clofibric Acid | - | 9.2 | 213.0324 | 126.9960 | Bezafibrate D4 |
| Cocaine | + | 10.5 | 304.1543 | 182.1178 | Cocaine D5 |
| Cocaethylene | + | 12 | 318.1700 | 196.1335 | Venlafaxine D6 |
| Codeine | + | 5.8 | 300.1594 | 165.0699 | Codeine D3 |
| Cotinine | + | 3 | 177.1022 | 80.0495 | Cotinine D3 |
| Crotamiton | + | 14.9 | 204.1383 | 69.0337 | Climbazole D4 |
| Cyclamate | - | 4.5 | 178.0543 | 79.9576 | Hydrochlorothiazide 13CD2 |
| Diazepam | + | 15 | 285.0789 | 193.0886 | Climbazole D4 |
| Diclofenac | + | 15.4 | 296.0240 | 215.0497 | Diclofenac 13C6 |
| Diltiazem | + | 13 | 415.1686 | 178.0321 | Carbamazepine D10 |
| Estriol | - | 11.8 | 287.1653 | 171.0820 | Bezafibrate D4 |
| Estrone | - | 14.9 | 269.1547 | 145.0664 | Ibuprofen D3 |
| Ezetimibe | - | 14.93 | 408.1417 | 271.1144 | Fipronil 13C3 |
| Fipronil | - | 16 | 434.9314 | 249.9588 | Fipronil 13C3 |
| Fipronil Desulfinyl | - | 16.1 | 386.9644 | 350.9882 | Fipronil 13C3 |
| Fipronil sulfide | - | 16.3 | 418.9365 | 313.9654 | Fipronil 13C3 |
| Fipronil sulfone | - | 16.3 | 450.9263 | 183.0179 | Fipronil 13C3 |
| Florfenicol | - | 10.8 | 355.9932 | 119.0506 | Furosemide D5 |
| Fluconazole | + | 9 | 307.1113 | 220.0681 | Fluconazole 13C3 |
| Flumequine | + | 13.6 | 262.0874 | 244.0768 | Carbamazepine D10 |
| Fluticasone propionate | + | 15.9 | 501.1917 | 313.1596 | Fluconazole 13C3 |
| Furazolidone | + | 9.2 | 226.0459 | 95.0366 | Fluconazole 13C3 |
| Furosemide | - | 9.8 | 329.0004 | 204.9848 | Furosemide D5 |
| Gemfibrozil | - | 15.9 | 249.1496 | 121.0663 | Gemfibrozil D6 |
| Hydrochlorothiazide | - | 6.3 | 295.9572 | 268.9467 | Hydrochlorothiazide 13CD2 |
| Hydroxychloroquine | + | 6 | 336.1837 | 247.0997 | Caffeine 13C3 |
| Hyoscine (scopolamine) | + | 11.1 | 360.2169 | 138.0914 | Cocaine D5 |
| Ibuprofen | - | 14.74 | 205.1234 | 159.1186 | Ibuprofen D3 |
| Indomethacin | - | 13.8 | 356.0695 | 297.0567 | Indomethacin D3/4 |
| Irbesartan | + | 14.5 | 429.2397 | 207.0918 | Ibersartan D6 |
| Irgasan (Triclosan) | - | 16.5 | 286.9439 | 118.9248 | Irgasan (Triclosan) 13C6 |
| Ketoprofen | + | 14.5 | 255.1016 | 105.0336 | Valsartan D3 |
| Lamotrigine | + | 9 | 256.0151 | 172.9670 | Lamotrigine 13C3 |
| Lidocaine | + | 8.2 | 235.1805 | 86.0965 | Benzotriazole D4 |
| Lincomycin | + | 6.3 | 407.2210 | 126.1276 | Caffeine 13C3 |
| Lamotrigine-N2-oxide | + | 8.8 | 272.0100 | 242.0122 | Lamotrigine 13C3 |
| Lorazepam | + | 13.82 | 321.0192 | 275.0138 | Lorazepam D4 |
| Losartan | + | 13.9 | 423.1695 | 207.0918 | Sulfamethoxazole 13C6 |
| Mefenamic Acid | + | 14.5 | 242.1176 | 224.1070 | Diclofenac 13C6 |
| Meprobamate | + | 11.1 | 219.1339 | 158.1176 | Carisoprodol D7 |
| Methadone | + | 13.8 | 310.2165 | 265.1588 | Carbamazepine D10 |
| Metoprolol | + | 9.4 | 268.1907 | 116.1070 | Metoprolol D7 |
| Metronidazole | + | 5.1 | 172.0717 | 128.0454 | Metronidazole D4 |
| Midazolam | + | 12.8 | 326.0855 | 291.1167 | Midazolam 13C6 |
| Morphine | + | 3.6 | 286.1438 | 201.0910 | Morphine D3 |
| N-acetyl sulfamethoxazole | + | 11.4 | 296.0700 | 134.0602 | Sulfamethoxazole 13C6 |
| Nalidixic acid | + | 13.45 | 233.0921 | 205.0607 | Carbamazepine D10 |
| Naproxen | - | 12.4 | 229.0870 | 158.0379 | Ibersartan D6 |
| Neotame | + | 13.1 | 379.2227 | 172.1332 | Carbamazepine D10 |
| N-Methyl lamotrigine | + | 10.4 | 270.0308 | 200.9979 | Lamotrigine 13C3 |
| O-desmethylvenlafaxine | + | 8.4 | 264.1958 | 246.1853 | Venlafaxine D6 |
| Oseltamivir | + | 11.8 | 313.2122 | 166.0863 | Oseltamivir D3 |
| Oxazepam | + | 13.6 | 287.0582 | 241.0528 | Lorazepam D4 |
| Paroxetine | + | 13.2 | 330.1500 | 192.1187 | Paroxetine D4 |
| Pentobarbital | - | 13.62 | 225.1245 | 183.1395 | Valsartan D3 |
| Phenytoin | - | 13.3 | 251.0826 | 102.0353 | Ibersartan D6 |
| Primidone | + | 8.7 | 219.1128 | 162.0916 | Sulfamethazine D4 |
| Propranolol | + | 12.4 | 260.1645 | 116.1070 | Propranolol D7 |
| Propyphenazone | + | 13.8 | 231.1492 | 189.1024 | Cotinine D3 |
| Rosuvastatin | + | 14 | 482.1756 | 258.1405 | Bezafibrate D4 |
| Saccharin | - | 3.8 | 181.9917 | 61.9705 | Hydrochlorothiazide 13CD2 |
| Salicylic acid | - | 4.45 | 137.0244 | 93.0349 | Hydrochlorothiazide 13CD2 |
| Sertraline | + | 13.9 | 306.0811 | 158.9763 | Sertraline D3 |
| Sitagliptin | + | 10.6 | 408.1254 | 174.0529 | Sitagliptin D4 |
| Sotalol | + | 4.6 | 273.1268 | 213.0694 | Sotalol D6 |
| Stevioside | - | 12.6 | 803.3707 | 641.3193 | 17b-Estradiol D5 |
| Sucralose | - | 7.5 | 395.0073 | 59.0137 | Sucralose D6 |
| Sulfadiazine | + | 5.9 | 251.0597 | 156.0114 | Sulfadiazine D4 |
| Sulfamerazine | + | 7.3 | 265.0754 | 156.0114 | Sulfamethazine D4 |
| Sulfamethazine | + | 8.4 | 279.0910 | 204.0440 | Sulfamethazine D4 |
| Sulfamethizole | + | 8.4 | 271.0318 | 156.0114 | Sulfamethazine D4 |
| Sulfamethoxazole | + | 10.6 | 254.0594 | 156.0114 | Sulfamethoxazole 13C6 |
| Sulfadimethoxine | + | 12.5 | 311.0809 | 156.0769 | Sulfamethoxazole 13C6 |
| Sulfapyridine | + | 6.9 | 250.0645 | 156.0114 | Sulfadiazine D4 |
| Sulfathiazole | + | 6.4 | 256.0209 | 156.0114 | Sulfamethazine D4 |
| Sulisobenzone (BP4) | - | 9.5 | 307.0282 | 211.0405 | Furosemide D5 |
| Tramadol | + | 9.5 | 264.1958 | 58.0654 | Tramadol 13C3D3 |
| Trimethoprim | + | 7.3 | 291.1452 | 230.1162 | Trimethoprim D9 |
| Valsartan | + | 14.6 | 436.2343 | 207.0918 | Valsartan D3 |
| Valsartan acid | + | 12.1 | 267.0877 | 206.0596 | Valsartan acid D4 |
| Venlafaxine | + | 11.6 | 278.2115 | 58.0654 | Venlafaxine D6 |
| Verapamil | + | 13.6 | 455.2904 | 165.0910 | Carbamazepine D10 |
| Warfarin | + | 15 | 309.1121 | 163.0389 | Climbazole D4 |
| Zonisamide | + | 9.6 | 213.0328 | 150.0550 | Zonisamide 13C6 |

**Table SM 6. Retention time, type of ionization and isotopically labelled compound associated of targeted compounds**

|  |  |  | **Intraday 5 ppb** | | **Intraday 50 ppb** | | **Intraday 500 ppb** | | **Interday 5 ppb** | | **Intreday 50 ppb** | | **Interday 500 ppb** | |
| --- | --- | --- | --- | --- | --- | --- | --- | --- | --- | --- | --- | --- | --- | --- |
| **Compound name** | **ESI** | **RT (min)** | **RR (%)** | **ME (%)** | **RR (%)** | **ME (%)** | **RR (%)** | **ME (%)** | **RR (%)** | **ME (%)** | **RR (%)** | **ME (%)** | **RR (%)** | **ME (%)** |
| 1,7 Dimethylxanthine | + | 4.9 | - | - | 140.70 | -52.21 | 113.69 | -69.17 | - | - | 126.79 | -38.34 | 101.66 | -60.90 |
| 17-α Ethynilestradiol | - | 14.7 | 54.97 | -38.05 | 51.39 | -43.17 | 57.68 | -45.65 | 52.98 | -39.46 | 49.57 | -46.33 | 56.43 | -47.64 |
| 1H-Benzotriazole | + | 7.4 | 59.91 | 7.91 | 70.41 | -50.80 | 81.90 | -56.02 | 58.68 | 26.73 | 73.13 | -46.78 | 82.33 | -53.50 |
| 2.4-Dihydroxybenzophenone (BP1) | - | 14.3 | 87.36 | -14.56 | 92.00 | -31.51 | 94.08 | -27.10 | 88.15 | -20.39 | 90.82 | -33.42 | 92.57 | -29.46 |
| 2.2'.4.4'-Tetrahydroxybenzophenone (BP2) | - | 12.5 | 92.53 | -65.31 | 95.89 | -79.92 | 88.76 | -62.60 | 94.86 | -68.12 | 95.19 | -80.55 | 87.65 | -63.59 |
| 4-Hydroxidiclofenac | + | 14.2 | 46.37 | -42.90 | 62.62 | -48.00 | 68.10 | -51.00 | 51.87 | -52.36 | 61.99 | -46.60 | 66.58 | -48.40 |
| 4-Nitro Sulfamethoxazole | - | 10 | 108.04 | 15.00 | 112.70 | 10.63 | 113.98 | 13.14 | 103.71 | 8.30 | 111.70 | 3.15 | 113.13 | 8.64 |
| 5-Desamino-5 oxo 2,5 Lamotrigine | + | 10.5 | 92.24 | -51.86 | 100.81 | -57.44 | 103.33 | -46.88 | 95.06 | -48.19 | 101.61 | -56.02 | 101.93 | -45.50 |
| 5-methyl-1 H Benzotriazole | + | 10 | 105.34 | 116.26 | 90.94 | -45.35 | 94.76 | -58.27 | 101.99 | 69.59 | 90.10 | -58.82 | 94.66 | -50.93 |
| 5-nitro-Diclofenac | - | 13.1 | 85.44 | -59.03 | 91.41 | -55.52 | 89.66 | -44.00 | 87.92 | -61.95 | 89.70 | -58.09 | 88.61 | -46.58 |
| 6a-Methylprednisolone | + | 13.3 | 99.77 | -57.93 | 118.62 | -82.62 | 98.33 | -71.73 | 104.15 | -54.85 | 116.13 | -81.24 | 97.32 | -70.71 |
| Acesulfame | - | 2.8 | 61.33 | >200 | 49.99 | 1.40 | 43.30 | -14.30 | 48.90 | >200 | 46.98 | 3.58 | 41.74 | -14.43 |
| Acetaminophen | + | 4.7 | 66.10 | -59.90 | 66.18 | -68.50 | 66.47 | -60.20 | 69.20 | -47.12 | 75.85 | -65.62 | 66.92 | -56.78 |
| Acridone | + | 12.6 | 107.85 | -78.59 | 89.56 | -76.70 | 90.17 | -76.13 | 107.93 | -79.09 | 90.76 | -77.57 | 89.90 | -76.87 |
| Adamantan-1-amine (Amantadine) | + | 7.6 | 95.35 | -43.52 | 97.45 | -53.79 | 101.89 | -59.28 | 94.22 | -37.72 | 99.38 | -52.24 | 100.30 | -57.67 |
| Alprazolam | + | 13.8 | 99.34 | -40.80 | 103.29 | -53.60 | 103.38 | -38.70 | 100.19 | -39.31 | 103.99 | -53.27 | 105.14 | -39.10 |
| Atenolol | + | 4.5 | 73.67 | -29.22 | 72.61 | -42.16 | 66.78 | -14.65 | 79.07 | -18.02 | 81.19 | -53.55 | 91.14 | -48.30 |
| Azithromycin | + | 10.9 | - | - | 25.09 | >200 | 108.99 | >200 | - | - | 25.81 | >200 | 110.02 | >200 |
| Benzoylecgonine | + | 7.8 | 109.15 | -52.62 | 110.46 | -68.13 | 112.97 | -59.51 | 109.50 | -50.02 | 109.92 | -67.70 | 113.62 | -59.37 |
| B-estradiol | - | 14.5 | 100.04 | -6.59 | 65.63 | -30.77 | 70.19 | -33.25 | 92.63 | -3.00 | 65.88 | -34.82 | 67.92 | -36.73 |
| Bezafibrate | + | 14.4 | 101.58 | -36.10 | 108.10 | -51.30 | 107.32 | -63.30 | 99.64 | -30.15 | 109.03 | -48.94 | 106.89 | -61.82 |
| Bisphenol-A | - | 9.5 | - | - | 77.15 | -38.22 | 72.13 | -38.80 | - | - | 77.51 | -41.95 | 71.88 | -41.40 |
| Bromazepam | + | 12.7 | 96.67 | 74.21 | 76.26 | 32.69 | 80.16 | -30.26 | 100.02 | 71.10 | 75.42 | 30.15 | 85.20 | -36.29 |
| Caffeine | + | 6.4 | - | - | 141.56 | -26.26 | 113.70 | -55.01 | - | - | 145.82 | -16.79 | 113.71 | -51.88 |
| Carazolol | + | 11.5 | 80.67 | >200 | 69.91 | >200 | 73.37 | 141.15 | 85.09 | >200 | 74.68 | >200 | 69.89 | 130.83 |
| Carbamazepine | + | 13.3 | 103.70 | -39.07 | 107.86 | -66.72 | 107.13 | -53.06 | 104.14 | -37.54 | 106.54 | -66.32 | 107.17 | -53.19 |
| Carbamazepine-10,11-epoxide | + | 12.0 | 109.46 | -46.38 | 91.89 | -42.90 | 96.70 | -51.57 | 106.35 | -50.50 | 90.12 | -45.64 | 96.60 | -53.14 |
| Carisoprodol | + | 13.9 | 96.32 | -48.40 | 111.22 | -58.30 | 111.48 | -49.80 | 100.52 | -45.16 | 108.29 | -55.66 | 110.90 | -48.27 |
| Chloramphenicol | - | 11.7 | 101.89 | -35.20 | 111.08 | -36.09 | 106.75 | -21.72 | 103.13 | -39.50 | 109.29 | -38.47 | 106.55 | -25.12 |
| Citalopram | + | 12.9 | 53.13 | 173.26 | 56.05 | -5.11 | 65.42 | -45.76 | 53.41 | 178.83 | 59.28 | 6.12 | 59.52 | -27.54 |
| Clarithromycin | + | 13.8 | 67.52 | -6.30 | 65.45 | -64.00 | 68.26 | -50.20 | 66.03 | -3.27 | 65.58 | -62.45 | 67.56 | -48.60 |
| Climbazole | + | 14.7 | 81.38 | -62.80 | 71.46 | -71.20 | 86.93 | -67.40 | 82.57 | -60.94 | 71.66 | -70.27 | 86.85 | -66.64 |
| Clofibric Acid | - | 9.2 | 116.95 | -6.05 | 113.31 | -30.49 | 109.00 | -24.07 | 114.01 | -3.36 | 111.98 | -27.61 | 109.07 | -20.14 |
| Cocaine | + | 10.5 | 74.86 | >200 | 79.75 | 133.92 | 86.30 | -19.80 | 76.10 | >200 | 79.65 | 140.97 | 87.02 | -19.53 |
| Cocaethylene | + | 12.0 | 88.49 | 35.05 | 78.81 | -8.97 | 84.09 | -21.15 | 89.48 | 28.31 | 78.70 | -11.82 | 83.82 | -23.03 |
| Codeine | + | 5.8 | 44.17 | -46.51 | 47.11 | -49.86 | 59.79 | -46.76 | 42.88 | -38.02 | 48.61 | -48.50 | 60.36 | -46.86 |
| Cotinine | + | 3.0 | 151.35 | 5.10 | 125.48 | -49.56 | 126.62 | -46.30 | 169.62 | 10.01 | 128.96 | -47.21 | 128.57 | -46.04 |
| Crotamiton | + | 14.9 | 44.19 | -81.00 | 26.31 | -85.00 | 30.73 | -79.20 | 44.14 | -79.35 | 26.75 | -84.23 | 30.52 | -78.34 |
| Cyclamate | - | 4.5 | 25.51 | 38.68 | 21.81 | -34.47 | 20.22 | -28.47 | 41.63 | 11.56 | 23.78 | -36.64 | 20.71 | -30.32 |
| Diazepam | + | 15 | 99.60 | -72.00 | 101.95 | -68.70 | 99.75 | -57.20 | 98.95 | -70.33 | 101.23 | -67.66 | 100.35 | -56.59 |
| Diclofenac | + | 15.4 | 55.91 | -67.70 | 78.75 | -62.10 | 81.77 | -66.60 | 64.18 | -62.47 | 77.98 | -60.80 | 80.87 | -64.94 |
| Diltiazem | + | 13 | 59.94 | 177.86 | 60.34 | 34.12 | 69.03 | -47.48 | 59.30 | 184.66 | 59.63 | 38.31 | 70.07 | -47.51 |
| Estriol | - | 11.8 | 84.73 | 51.77 | 89.07 | -22.58 | 88.41 | -19.08 | 106.98 | 46.45 | 86.95 | -29.07 | 85.33 | -26.63 |
| Estrone | - | 14.9 | 76.67 | 1.04 | 72.66 | -27.75 | 73.70 | -35.54 | 81.51 | -5.51 | 70.90 | -31.30 | 71.54 | -37.89 |
| Ezetimibe | - | 14.9 | 47.91 | 39.84 | 25.71 | -12.74 | 37.09 | -68.73 | 45.86 | 32.25 | 25.50 | -16.04 | 35.42 | -68.88 |
| Fipronil | - | 16.0 | 63.04 | -10.24 | 38.74 | -20.60 | 58.79 | -17.46 | 63.72 | -16.05 | 39.18 | -24.55 | 57.26 | -18.77 |
| Fipronil desulfinyl | - | 16.1 | 65.37 | -56.68 | 36.68 | -57.87 | 57.59 | -46.47 | 63.87 | -58.43 | 36.59 | -59.50 | 55.93 | -46.64 |
| Fipronil sulfide | - | 16.3 | 51.92 | -71.27 | 31.79 | -73.08 | 51.44 | -58.70 | 52.62 | -72.68 | 31.93 | -74.50 | 49.52 | -58.55 |
| Fipronil sulfone | - | 16.3 | 52.32 | -73.57 | 30.16 | -76.95 | 49.50 | -67.73 | 51.33 | -74.54 | 30.44 | -78.02 | 46.18 | -66.22 |
| Florfenicol | - | 10.8 | 100.74 | -36.23 | 110.28 | -25.02 | 105.47 | -16.20 | 100.57 | -41.17 | 109.33 | -28.44 | 105.22 | -19.62 |
| Fluconazole | + | 9.0 | 103.22 | -41.88 | 108.17 | -52.37 | 110.89 | -59.33 | 103.34 | -32.70 | 108.11 | -48.39 | 110.52 | -56.78 |
| Flumequine | + | 13.6 | 90.24 | -51.76 | 93.72 | -66.79 | 97.10 | -64.97 | 90.04 | -49.80 | 92.57 | -66.23 | 97.78 | -64.39 |
| Fluticasone propionate | + | 15.9 | 53.36 | -82.70 | 28.82 | -82.80 | 45.07 | -85.00 | 71.50 | -81.18 | 54.57 | -81.65 | 82.49 | -83.94 |
| Furazolidone | + | 9.2 | 110.73 | -69.68 | 121.04 | -74.90 | 110.72 | -69.07 | 106.75 | -63.10 | 120.65 | -71.50 | 111.97 | -66.78 |
| Furosemide | - | 9.8 | 55.42 | -40.62 | 72.45 | -51.76 | 80.81 | -44.91 | 61.95 | -44.60 | 73.40 | -53.09 | 80.02 | -45.47 |
| Gemfibrozil | - | 15.9 | 46.38 | -41.53 | 45.61 | -68.37 | 54.93 | >200 | 46.86 | -43.41 | 44.74 | -68.75 | 53.43 | >200 |
| Hydrochlorothiazide | - | 6.3 | 180.58 | 25.48 | 111.91 | -50.50 | 104.07 | -43.99 | 174.47 | 17.76 | 108.57 | -51.48 | 103.70 | -46.14 |
| Hydroxychloroquine | + | 6.0 | 29.63 | >200 | 50.72 | >200 | 60.44 | >200 | 32.37 | >200 | 52.03 | >200 | 64.07 | >200 |
| Hyoscine (scopolamine) | + | 11.1 | 102.06 | -36.50 | 106.09 | -54.40 | 102.65 | -58.90 | 101.09 | -34.05 | 104.95 | -52.63 | 103.09 | -58.26 |
| Ibuprofen | - | 14.7 | - | - | 180.72 | 15.58 | 97.26 | -37.88 | - | - | 174.13 | 14.10 | 93.99 | -36.42 |
| Indomethacin | - | 13.8 | 66.22 | -61.76 | 61.06 | -61.76 | 61.62 | -59.95 | 63.83 | -62.81 | 59.64 | -61.35 | 60.65 | -58.81 |
| Irbesartan | + | 14.5 | 34.61 | -67.30 | 43.34 | -78.60 | 58.65 | -75.80 | 36.82 | -64.86 | 43.39 | -77.00 | 58.45 | -74.46 |
| Irgasan (Triclosan) | - | 16.5 | 20.57 | -67.41 | 20.42 | -87.24 | 45.78 | -85.59 | 20.79 | -67.61 | 22.69 | -87.65 | 42.90 | -84.35 |
| Ketoprofen | + | 14.5 | 97.63 | 32.10 | 108.59 | -41.80 | 104.57 | -59.40 | 94.24 | 36.79 | 107.88 | -38.33 | 105.43 | -58.59 |
| Lamotrigine | + | 9.0 | 96.48 | -72.74 | 96.04 | -78.41 | 101.57 | -69.20 | 93.50 | -69.56 | 95.47 | -77.29 | 101.23 | -68.11 |
| Lamotrigine-N2-oxide | + | 8.2 | 86.42 | -67.58 | 92.71 | -71.31 | 88.20 | -61.96 | 85.99 | -65.41 | 92.40 | -70.09 | 88.65 | -61.07 |
| Lidocaine | + | 6.3 | 38.16 | -48.86 | 35.97 | -58.53 | 55.89 | -52.60 | 38.60 | -48.18 | 36.00 | -58.13 | 55.94 | -52.68 |
| Lincomycin | + | 8.8 | 48.83 | -0.99 | 57.66 | -14.43 | 46.71 | -55.12 | 40.68 | 27.26 | 56.69 | -44.31 | 50.84 | -37.74 |
| Lorazepam | + | 13.8 | 98.41 | -24.30 | 105.08 | -37.20 | 102.31 | -40.40 | 98.23 | -19.87 | 104.11 | -34.74 | 100.07 | -37.38 |
| Losartan | + | 13.9 | 18.72 | -48.00 | 37.05 | -64.60 | 47.37 | -61.40 | 19.89 | -45.17 | 37.44 | -63.20 | 47.27 | -59.96 |
| Mefenamic Acid | + | 14.5 | 56.03 | -72.50 | 55.98 | -82.50 | 48.67 | -80.70 | 59.36 | -72.22 | 54.64 | -81.65 | 49.15 | -80.13 |
| Meprobamate | + | 11.1 | 106.69 | -50.28 | 116.66 | -45.64 | 111.14 | -43.33 | 107.73 | -42.30 | 116.63 | -40.20 | 112.28 | -40.22 |
| Methadone | + | 13.8 | 56.37 | >200 | 57.57 | 9.50 | 63.74 | -18.80 | 56.80 | >200 | 57.24 | 11.75 | 63.91 | -18.27 |
| Metoprolol | + | 9.4 | 43.21 | -10.85 | 63.17 | -45.87 | 70.60 | -50.87 | 42.80 | -6.45 | 63.14 | -44.47 | 70.65 | -50.33 |
| Metronidazole | + | 5.1 | 78.47 | -58.27 | 86.42 | -61.34 | 85.76 | -63.14 | 82.22 | -49.53 | 88.06 | -58.26 | 82.87 | -59.33 |
| Midazolam | + | 12.8 | 64.96 | -75.34 | 66.60 | -75.94 | 57.79 | -75.53 | 65.34 | -75.66 | 65.86 | -76.49 | 57.35 | -75.84 |
| Morphine | + | 3.6 | 47.94 | -56.95 | 50.15 | -67.75 | 61.41 | -57.21 | 47.40 | -46.98 | 50.65 | -63.49 | 62.84 | -54.37 |
| N-acetyl sulfamethoxazole | + | 11.4 | 101.71 | -40.72 | 107.44 | -45.85 | 107.08 | -54.85 | 100.91 | -33.87 | 109.14 | -41.76 | 107.04 | -52.81 |
| Nalidixic acid | + | 13.4 | 82.38 | -63.80 | 86.72 | -71.12 | 94.03 | -61.00 | 84.32 | -63.09 | 85.76 | -70.71 | 94.15 | -60.41 |
| Naproxen | - | 12.4 | - | - | 136.99 | -49.79 | 110.00 | -53.66 | - | - | 122.86 | -42.84 | 105.90 | -50.27 |
| Neotame | + | 13.1 | 66.60 | 61.86 | 93.22 | 48.71 | 96.19 | -38.91 | 73.75 | 53.10 | 92.53 | 53.77 | 97.35 | -37.90 |
| N-Methyl lamotrigine | + | 10.4 | 99.92 | -25.72 | 105.99 | -51.18 | 104.46 | -49.95 | 101.61 | -22.13 | 105.23 | -49.51 | 103.64 | -48.75 |
| O-desmethylvenlafaxine | + | 8.4 | 67.49 | -31.01 | 70.39 | -61.64 | 80.06 | -51.57 | 68.47 | -30.78 | 70.76 | -61.11 | 80.51 | -50.82 |
| Oseltamivir | + | 11.8 | 73.34 | 22.60 | 64.47 | 32.48 | 72.76 | -4.77 | 74.07 | 11.52 | 63.59 | 30.43 | 72.23 | -5.74 |
| Oxazepam | + | 13.6 | 100.00 | -34.80 | 111.61 | -45.70 | 104.35 | -46.00 | 99.96 | -30.88 | 112.67 | -43.45 | 104.07 | -42.71 |
| Paroxetine | + | 13.2 | 18.69 | >200 | 27.32 | >200 | 35.35 | 87.05 | 17.14 | >200 | 26.32 | >200 | 35.70 | 94.46 |
| Pentobarbital | - | 13.6 | 102.66 | -40.48 | 114.66 | -41.87 | 112.10 | -31.57 | 103.21 | -41.59 | 116.16 | -42.24 | 110.41 | -29.81 |
| Phenytoin | - | 13.3 | 99.88 | -37.83 | 105.81 | -44.44 | 105.66 | -35.14 | 101.50 | -38.82 | 106.37 | -44.90 | 103.78 | -34.60 |
| Primidone | + | 8.7 | - | - | 85.86 | -24.39 | 98.84 | -54.74 | - | - | 93.11 | -28.13 | 97.06 | -52.82 |
| Propranolol | + | 12.4 | 80.05 | 126.03 | 68.30 | 32.22 | 74.71 | -17.11 | 86.04 | 121.76 | 70.65 | 29.05 | 74.25 | -19.03 |
| Propyphenazone | + | 13.8 | 100.49 | -73.80 | 103.57 | -75.00 | 106.26 | -54.20 | 100.55 | -73.13 | 101.95 | -74.93 | 106.33 | -54.92 |
| Rosuvastatin | + | 14 | 99.86 | -42.20 | 105.06 | -44.20 | 100.87 | -52.60 | 99.39 | -41.28 | 105.03 | -43.06 | 101.35 | -50.86 |
| Saccharin | - | 3.8 | 150.00 | >200 | 53.94 | 107.70 | 102.01 | -7.23 | 140.00 | >200 | 47.81 | 95.21 | 104.36 | -10.75 |
| Salicylic acid | - | 4.4 | 149.69 | >200 | 128.79 | 10.50 | 107.37 | -50.30 | 130.60 | >200 | 115.69 | 8.06 | 114.25 | -58.75 |
| Sertraline | + | 13.9 | 36.31 | >200 | 26.83 | >200 | 38.11 | >200 | 37.89 | >200 | 27.20 | >200 | 37.53 | >200 |
| Sitagliptin | + | 10.6 | 37.87 | 2.14 | 47.05 | -32.99 | 49.72 | -51.45 | 44.25 | 2.04 | 47.12 | -30.75 | 49.57 | -50.30 |
| Sotalol | + | 4.6 | 73.77 | -46.79 | 85.24 | -58.60 | 93.11 | -54.88 | 76.30 | -38.11 | 86.64 | -53.57 | 93.84 | -51.64 |
| Stevioside | - | 12.6 | - | - | 104.65 | -42.86 | 98.36 | -31.61 | - | - | 102.42 | -45.97 | 96.97 | -36.62 |
| Sucralose | - | 7.5 | - | - | 134.63 | 52.36 | 111.71 | -22.16 | - | - | 134.79 | 40.23 | 107.28 | -26.58 |
| Sulfadiazine | + | 5.9 | 81.74 | -1.09 | 65.36 | -9.04 | 77.76 | -43.27 | 81.34 | 6.19 | 66.05 | -6.22 | 78.93 | -42.77 |
| Sulfamerazine | + | 7.3 | 85.68 | -12.61 | 71.91 | -16.68 | 67.93 | -40.56 | 86.32 | -8.23 | 72.76 | -23.73 | 78.21 | -40.07 |
| Sulfamethazine | + | 8.4 | 85.23 | -12.08 | 70.15 | -25.41 | 81.24 | -36.02 | 86.36 | -9.01 | 69.66 | -22.69 | 82.21 | -35.61 |
| Sulfamethizole | + | 8.4 | 72.53 | -49.11 | 60.93 | -44.96 | 73.27 | -56.62 | 73.72 | -46.21 | 60.70 | -42.77 | 73.92 | -55.85 |
| Sulfamethoxazole | + | 10.6 | 52.37 | 5.46 | 74.60 | -42.71 | 85.18 | -58.51 | 51.90 | 16.00 | 74.59 | -38.66 | 85.45 | -56.94 |
| Sulfamethoxine (Sulfadimethoxine) | + | 12.5 | 116.84 | -76.45 | 82.97 | -62.06 | 91.51 | -67.05 | 118.20 | -77.64 | 83.15 | -63.99 | 90.55 | -68.23 |
| Sulfapyridine | + | 6.9 | 81.02 | -32.56 | 66.88 | -30.38 | 78.70 | -48.21 | 81.26 | -26.03 | 67.48 | -26.26 | 79.43 | -46.70 |
| Sulfathiazole | + | 6.4 | 74.68 | 8.50 | 63.45 | -36.12 | 79.34 | -54.12 | 74.71 | 12.23 | 64.02 | -34.48 | 79.80 | -53.73 |
| Sulisobenzone (BP4) | - | 14.2 | 87.22 | -47.63 | 99.23 | -47.80 | 89.37 | -39.44 | 84.46 | -49.54 | 99.41 | -49.08 | 89.41 | -40.50 |
| Tramadol | + | 9.5 | 42.23 | -34.82 | 51.65 | -58.32 | 65.89 | -57.07 | 43.16 | -32.06 | 51.06 | -57.40 | 65.34 | -56.30 |
| Trimethoprim | + | 7.3 | 90.66 | -58.02 | 96.22 | -63.78 | 97.03 | -56.07 | 90.73 | -54.66 | 96.75 | -61.89 | 97.93 | -54.96 |
| Valsartan | + | 14.6 | 64.86 | 20.30 | 88.14 | -47.60 | 88.09 | -48.20 | 62.49 | 32.20 | 84.95 | -42.80 | 87.74 | -46.08 |
| Valsartan acid | + | 12.1 | 53.91 | -22.57 | 80.68 | -31.83 | 89.62 | -37.72 | 78.62 | -30.40 | 80.79 | -29.52 | 89.88 | -35.82 |
| Venlafaxine | + | 11.6 | 75.43 | -31.70 | 65.98 | -41.59 | 73.77 | -53.44 | 72.84 | -35.33 | 66.11 | -44.94 | 72.87 | -54.76 |
| Verapamil | + | 13.6 | 50.75 | >200 | 46.94 | 58.70 | 51.58 | -32.50 | 49.97 | >200 | 46.59 | 63.19 | 51.92 | 31.37 |
| Warfarin | + | 15.0 | 87.27 | -65.10 | 92.00 | -71.90 | 83.07 | -59.70 | 89.94 | -65.05 | 91.54 | -69.82 | 83.47 | -58.27 |
| Zonisamide | + | 9.6 | - | - | 122.28 | -48.29 | 114.81 | -42.25 | - | - | 116.01 | -47.80 | 113.84 | -43.22 |
